# A motif within the armadillo repeat of Parkinson’s-linked LRRK2 interacts with FADD to hijack the extrinsic death pathway

**DOI:** 10.1038/s41598-018-21931-8

**Published:** 2018-02-22

**Authors:** Nasia Antoniou, Dimitrios Vlachakis, Anna Memou, Emmanouela Leandrou, Polytimi-Eleni Valkimadi, Katerina Melachroinou, Diane B. Re, Serge Przedborski, William T. Dauer, Leonidas Stefanis, Hardy J. Rideout

**Affiliations:** 1 Division of Basic Neurosciences, Biomedical Research Foundation of the Academy of Athens, Athens, Greece; 2 Computational Biology, Biomedical Research Foundation of the Academy of Athens, Athens, Greece; 3 Department of Environmental Health Sciences, Columbia University, New York, NY USA; 4 Department of Neurology/Motor Neuron Center, Columbia University, New York, NY USA; 5 Department of Cell and Developmental Biology, University of Michigan Medical School, Ann Arbor, MI USA; 6 Second Department of Neurology, University of Athens Medical School, Athens, Greece

## Abstract

In experimental models, both *in vivo* and cellular, over-expression of Parkinson’s linked mutant leucine-rich repeat kinase 2 (LRRK2) is sufficient to induce neuronal death. While several cell death associated proteins have been linked to LRRK2, either as protein interactors or as putative substrates, characterization of the neuronal death cascade remains elusive. In this study, we have mapped for the first time the domain within LRRK2 that mediates the interaction with FADD, thereby activating the molecular machinery of the extrinsic death pathway. Using homology modeling and molecular docking approaches, we have identified a critical motif within the N-terminal armadillo repeat region of LRRK2. Moreover, we show that co-expression of fragments of LRRK2 that contain the FADD binding motif, or deletion of this motif itself, blocks the interaction with FADD, and is neuroprotective. We further demonstrate that downstream of FADD, the mitochondrial proteins Bid and Bax are recruited to the death cascade and are necessary for neuronal death. Our work identifies multiple novel points within neuronal death signaling pathways that could potentially be targeted by candidate therapeutic strategies and highlight how the extrinsic pathway can be activated intracellularly in a pathogenic context.

## Introduction

Expression of mutant forms of the Parkinson’s disease (PD) associated leucine-rich repeat kinase 2 (LRRK2) in primary neurons or neuronal-like cell lines induces death of a predominantly apoptotic form^1–4^. While the loss of substantia nigra (SN) dopaminergic neurons *in vivo* is likely to be mediated by additional factors, including critical non-cell autonomous mediators potentially triggered by degenerating neurons; understanding the precise neuronal signaling that underlies mutant LRRK2-induced neurotoxicity provides the opportunity for unraveling the still elusive roles and interactors of this protein, while developing novel therapeutic options. The known pathogenic mutations spanning the different enzymatic cores of LRRK2 elicit distinct changes in the function of LRRK2; however the different mutations induce neuronal death to a remarkably similar extent, sharing a reliance on kinase activity as well as activation of FADD-dependent death pathways^1,2,5,6^.

While a baseline interaction exists between wild type LRRK2 and FADD, even at endogenous levels in mouse brain, the interaction is strengthened, in a kinase-dependent fashion, by PD-linked mutations in LRRK2^1^. We have observed this also with other rare LRRK2 sequence variants that similarly induce apoptotic death of primary neurons^7^. Down-regulation of the upstream caspase-8, or blocking the ability of endogenous FADD to recruit caspase-8, prevents apoptotic death induced by mutant LRRK2. Importantly, activated caspase-8 is detected in SN of mutant G2019S-LRRK2 transgenic mice^8^, as well as in post-mortem brain tissue from PD patients carrying the G2019S or R1441C mutations in LRRK2^1^. Thus, while some elements of the extrinsic death pathway appear to be activated by mutant LRRK2, as well as the late-stage effector caspase-3^8,9^; the mediators of this activation are unknown, as is the mechanism by which FADD is activated.

The adaptor protein FADD contains a “death domain” (DD), which binds the cytoplasmic tail of death receptors as well as LRRK2^1^; and a “death effector domain” (DED), which recruits and binds caspase-8^10^. However it is not known what region of LRRK2 interacts with FADD. Here we report that a motif within the armadillo repeat region of the LRRK2 N-terminal domain is required for the interaction between LRRK2 and FADD, and that this motif is essential for the induction of apoptotic neuronal death induced by mutant forms of LRRK2 associated with PD. We have modeled *in silico* the structural basis for this interaction that is fully supported by our biochemical evidence. In addition to the activation of FADD and caspase-8, we demonstrate recruitment of key mitochondrial pro-apoptotic proteins, Bid and Bax, in the neuronal death pathway induced by mutant LRRK2. Taken together, our findings clearly define the molecular signaling of mutant LRRK2 downstream of FADD, recruiting elements of the intrinsic mitochondrial death machinery in primary neurons to induce death. The structural model of the interaction between LRRK2 and FADD we have created can serve as a basis for the rational design of novel therapeutic strategies aimed at interrupting mutant LRRK2-induced neuronal death.

## Results

### Inhibition of Bax signaling blocks mutant LRRK2-induced apoptotic death

**Figure 1:**
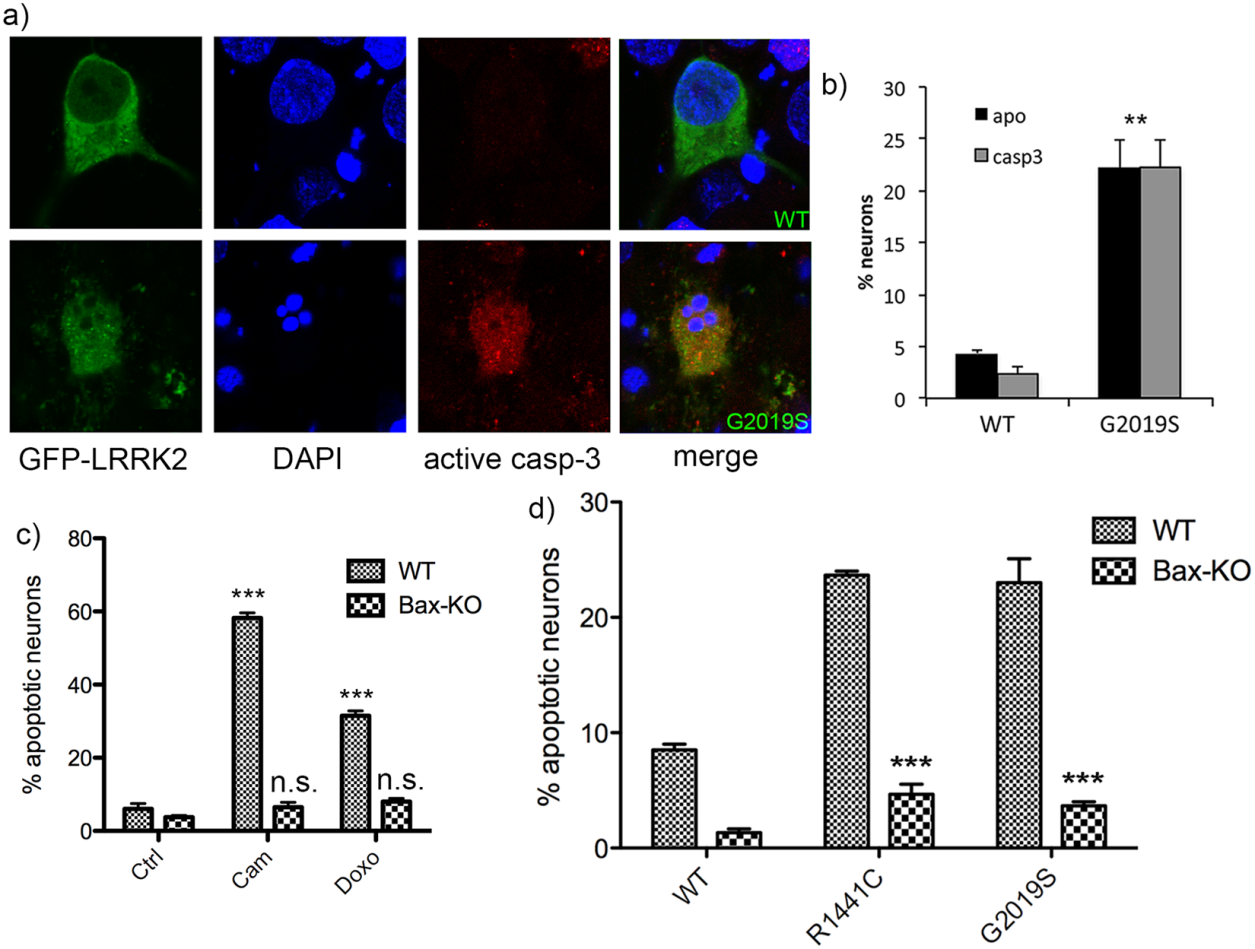
Genetic deletion of Bax prevents mutant LRRK2 induced neuronal death. Primary embryonic cortical neurons are derived from WT mice or mice lacking the pro-apoptotic protein Bax, and transiently transfected with GFP-tagged WT or mutant LRRK2 (R1441C, G2019S). (**a**) A representative image showing typical morphological features of apoptotic neurons expressing mutant LRRK2; that are positive for active caspase-3 (casp3) and display condensed and fragmented nuclei. (**b**) Quantification of apoptotic neuronal death (apoptotic nuclear profiles, “apo”; and active caspase-3 positive neurons, “casp3”) from cultures depicted in (**a**); **p < 0.01 in comparison to WT transfected neurons. (**c**) Primary neurons were prepared from WT mice or mice deficient in Bax, and treated with the topoisomerase I inhibitor camptothecin (“cam”, 5 μM, 18 hr), or doxorubicin (“doxo”, 10 ng/ml, 36 hr). Cells were fixed and processed for apoptotic nuclear counts. (**d**) Primary cortical neurons were prepared from WT mice or mice deficient in Bax, and transiently transfected with WT, R1441C, or G2019S LRRK2. As before, the cultures were fixed and the percentage of LRRK2-positive neurons that displayed apoptotic nuclear features was determined. The absence of Bax significantly protected neurons from death induced by mutant LRRK2. ***p < 0.001 compared to WT neurons expressing R1441C- or G2019S-LRRK2. Each data point represents the mean +/− SEM from 3–4 independent transfections from a representative culture. Cultures were repeated at least 3 times with similar differences.

To begin to characterize the downstream death signaling events following activation of extrinsic pathway components by mutant LRRK2, we asked whether the recruitment of intrinsic mitochondrial death-associated proteins Bid and Bax occurred. Multiple groups have shown that transient over-expression of mutant LRRK2 in purified primary neurons is sufficient to induce apoptotic death involving the activation of downstream effector caspase-3^9^. A representative image of neurons expressing WT or G2019S-LRRK2 at 48 h following transfection is shown in Fig. 1(a), and quantified in (b). As expected, neurons harboring apoptotic morphological changes also show active caspase-3 staining (Fig. 1a, red). We prepared embryonic cortical neurons from mice lacking Bax^11^ or control neurons from WT littermates, which were transiently transfected with mutant LRRK2. In parallel, to characterize these neurons, Bax-deficient cultures were treated with either the DNA topoisomerase inhibitor camptothecin^12^, or the chemotherapeutic agent doxorubicin. This toxin has been shown in multiple cells types to activate Fas-dependent cell death independently of the plasma membrane receptor^13,14^. Neurons derived from Bax-deficient mice are resistant to apoptotic death induced by either toxin (Fig. 1c). Importantly, we find that neurons lacking Bax are also resistant to the induction of apoptotic death triggered by over-expression of mutant LRRK2 (Fig. 1d). This suggests that intrinsic neuronal death mechanisms are also activated by mutant LRRK2.

**Figure 2:**
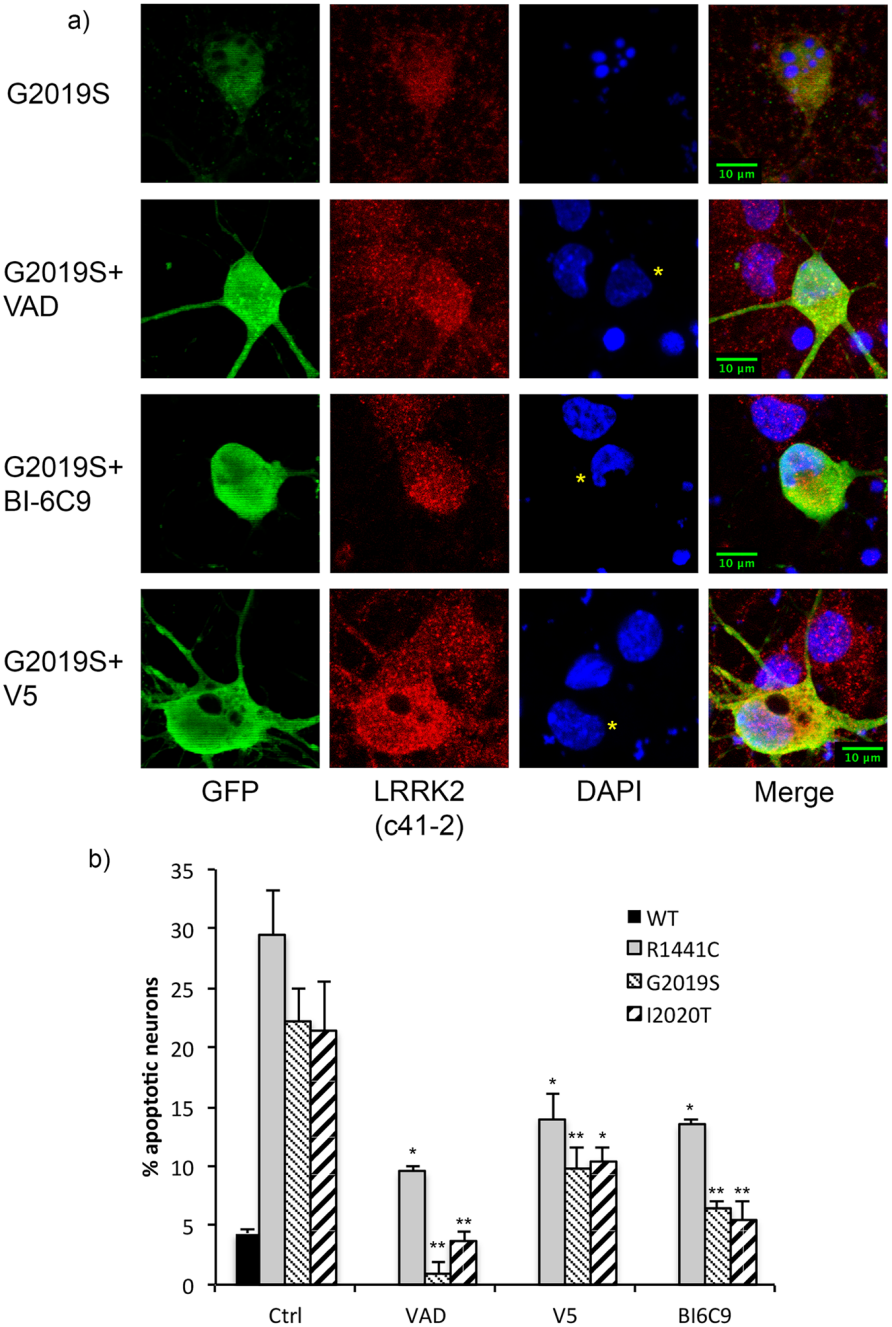
Pharmacological inhibition of Bax and Bid block apoptotic death induced by mutant LRRK2. WT cortical neurons transiently expressing WT, R1441C, G2019S, or I2020T LRRK2 (with an EGFP reporter) were vehicle treated, or treated with a pan caspase inhibitor (VAD), or peptide inhibitors of Bax (V5) or Bid (BI-6C9). (**a**) A representative confocal image of G2019S mutant LRRK2 expressing neurons, treated with vehicle (DMSO), VAD, V5, or BI-6C9. The percentage of neurons positive for the EGFP reporter and anti-LRRK2 (clone c41–2) exceeded 85%. The stained neurons were counted in a blinded fashion, and the percentage of GFP-positive neurons with apoptotic nuclear features was determined. Inhibition of caspase activation, as well as translocation of Bax or truncated Bid to the mitochondria, protects neurons from apoptotic death induced by different mutant forms of LRRK2. *p < 0.05 compared to untreated R1441C-LRRK2 expressing neurons; **p < 0.01 compared to untreated G2019S or I2020T-LRRK2 expressing neurons. Each data point represents the mean +/− SEM from 3–4 independent transfections from a representative culture. Cultures were repeated at least 3 times with similar differences. Asterisks indicate LRRK2-expressing neurons.

We next asked if pharmacological inhibition of the death signaling axis connecting caspase-8 and intrinsic death molecules could also block mutant LRRK2-induced neuronal death. WT cortical neurons transiently over-expressing mutant LRRK2 were then treated with vehicle or Bax and Bid inhibitory peptides, or a pan-caspase inhibitor as an additional control. In contrast to vehicle treated neurons, those treated with the Bax-inhibiting peptide V5^15^, the Bid-inhibiting peptide BI-6C9^16^, or the pan caspase inhibitor zVAD, were protected from apoptotic death induced by multiple mutant forms of LRRK2 (Fig. 2a,b). Representative images of mutant G2019S-expressing neurons are shown in Fig. 2(a), and neurons expressing R1441C- or I2020T-LRRK2 are shown in Suppl Fig. 1. Taken together, these data further characterize the cell death-associated signaling events triggered by expression of mutant LRRK2 in neurons; the activation of FADD/caspase-8 extrinsic death components followed by the recruitment of intrinsic mitochondrial death mediators Bid and Bax, and provide an expanded context for the late-stage participation of caspase-3 in neurons expressing mutant LRRK2.

### A region within the N-terminal domain of LRRK2 mediates the interaction with FADD

**Figure 3:**
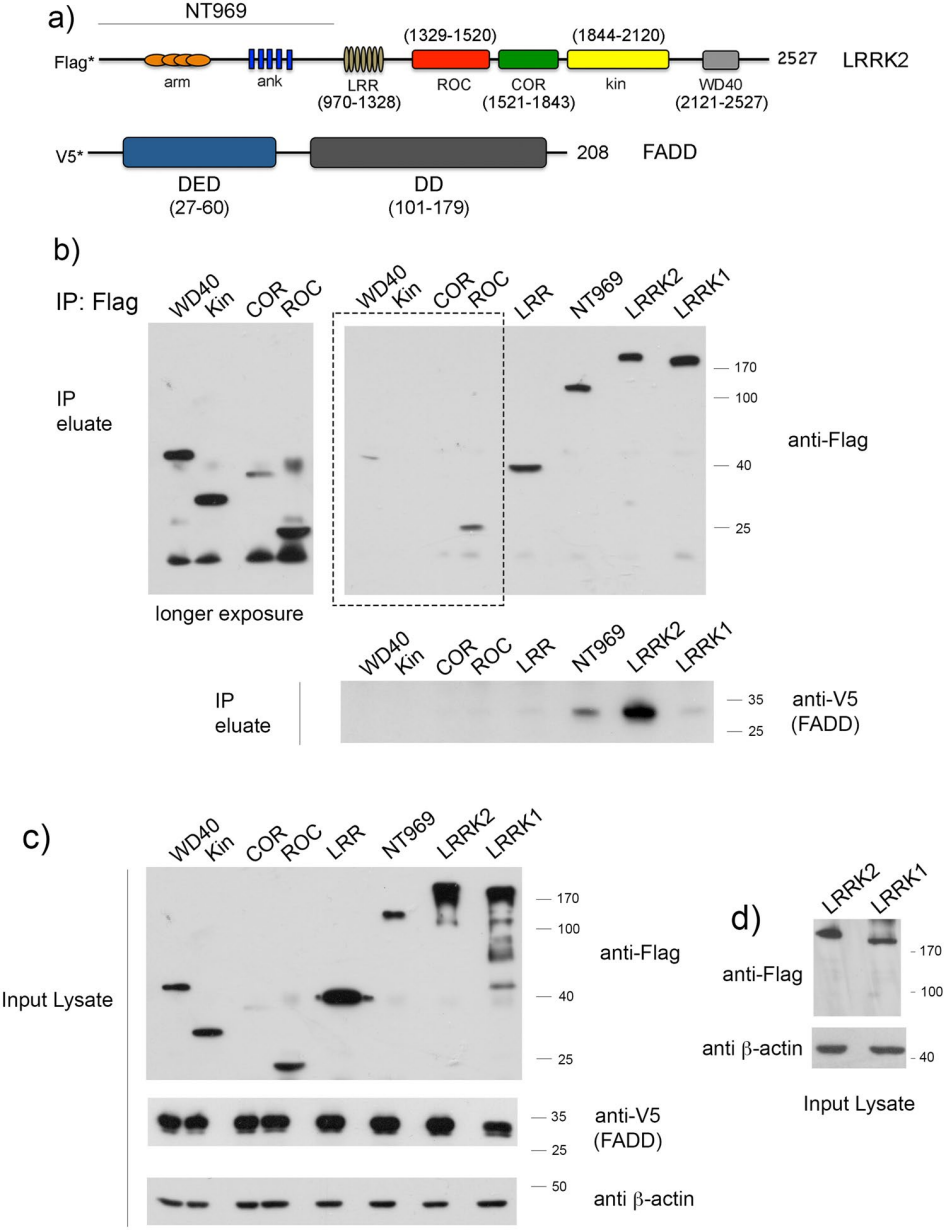
The N-terminal region upstream of the LRRK2 leucine-rich repeat interacts with FADD. (**a**) A schematic of the domain structure of LRRK2 with amino acid boundaries used in this study to map the interacting domain with FADD (lower schematic). (**b**) V5-tagged FADD was co-expressed in HEK293T cells with Flag-tagged LRRK1, or Flag-tagged constructs of LRRK2, encoding the full-length protein, or each of the discrete domains as illustrated in (**a**). Cell lysates were incubated with anti-Flag resin and the inputs and eluates were separated by 12% SDS-PAGE, followed by probing the membranes with anti-V5, anti-Flag, or anti-β-actin. The upper blots show the immunoprecipitation of Flag-LRRK1 or Flag-LRRK2 and probed for anti-Flag, and the co-precipitation of V5-FADD is shown in the middle panel (IP eluate) probed with anti-V5. FADD interacts with full-length LRRK2, but not the related LRRK1, and binds only the N-terminal domain upstream of the LRR. The left blot (in panel [b]) shows a longer exposure of the region highlighted by the box. Identical amounts of protein from the input lysate from each sample was separated by 12% SDS-PAGE, and the membranes probed with anti-Flag, anti-V5, and anti β-actin (**c**). In the blot shown in (**d**), identical lysates from cells expressing Flag-LRRK1 or Flag-LRRK2 were separated by 8% SDS-PAGE, to allow better separation of the LRRK1 and LRRK2 proteins.

To map the region of LRRK2 that binds FADD, we co-expressed V5-tagged FADD together with Flag-tagged domains of LRRK2 (as indicated in the schematic in Fig. 3a). Full-length WT LRRK2 associates with FADD (Fig. 3b) via its death domain (DD), as was shown previously^1^, but the closely related LRRK1 shows no significant interaction (Fig. 3b). A parallel immunoblot probed with anti-Flag is shown, from the same lysates separated by 8% SDS-PAGE, rather than 12%, to allow better appreciation of the difference in relative molecular mass between LRRK1 and LRRK2 (Fig. 3d). The slight V5-reactive band occasionally detected in the eluate of LRRK1-expressing cells may be indirect, via the interaction of over-expressed LRRK1 and FADD with endogenous LRRK2, as LRRK1 is a known interactor of LRRK2^17^. We find that only the N-terminal region upstream of the LRR domain, comprised of armadillo and ankyrin repeats, interacts strongly with FADD (Fig. 3b). As with LRRK1, faint V5-reactive bands were sometimes observed in cells expressing the ROC or COR domains may also be indirect due to interactions with endogenous LRRK2, which have been proposed to occur through this domain tandem^18^. Collectively, the evidence supports a mode of interaction occurring between the DD of FADD and the N-terminal domain of LRRK2.

**Figure 4:**
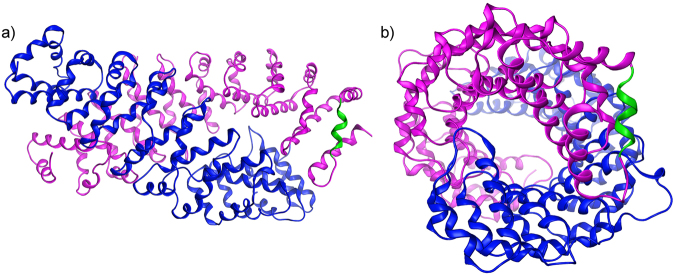
The 3D homology model of the dimeric LRRK2 ARM domain. (**a**) The armadillo repeats of the two LRRK2 molecules establish a concave inner conformation to each monomer. In this model of the isolated ARM repeat domain, the sites capable of dimerization are lined with residues that establish a hydrophobic core. Shown are each of the modeled LRRK2 monomers (presented in blue and magenta ribbon) of the LRRK2 ARM region. Each structure bears a specific α-helical motif that is exposed on the molecule located near the C-terminus of the ARM repeat region. One such motif is highlighted in green. (**b**) When viewed from the side, the two monomers display a supercoiled dimeric-like formation that is typical of armadillo repeat containing proteins.

To further refine the precise region of LRRK2 mediating the interaction with FADD, we undertook an *in silico* modeling approach. In the absence of a solved crystal structure of this region, we created a structural model of this domain to serve as a guide for identifying candidate residues that bind FADD. The model of a region within the LRRK2 armadillo (ARM) repeat domain was designed using conventional homology modeling techniques with the human Importin-alpha1 (PDB id: 3WPT^19^) crystal structure as a template. The model exhibited a rms deviation of atomic positions of less than 2 Å when structurally superposed to its template after energy minimization and MD simulations. Procheck scores and the corresponding Ramachandran plots confirmed that the 3D packing quality of the model is similar to the template crystal structure (Suppl Fig. 2). The resultant model of the LRRK2 ARM region predicts an anti-parallel arrangement when assembled as a dimer (Fig. 4a), similar to that predicted by recent homology modeling of the full length LRRK2 model^20,21^. The N-terminal region of LRRK2, when over-expressed, can also form dimers and oligomers^22^, which may serve to partly stabilize full-length LRRK2 dimers. The N-terminal part of the ARM model is comprised primarily of repeating helix-loop-helix motifs. However, approximately one-third towards the C-terminus of the model, the exposed helix introduces a kink that progressively interrupts that α-helix into two shorter ones. This conformation is essential to the stability of the dimer in this model, as stronger triangular shaped repeats are formed that are significantly more robust than the initial helix-loop-helix formation.

### Docking of the FADD DD to the LRRK2 ARM repeat

**Figure 5:**
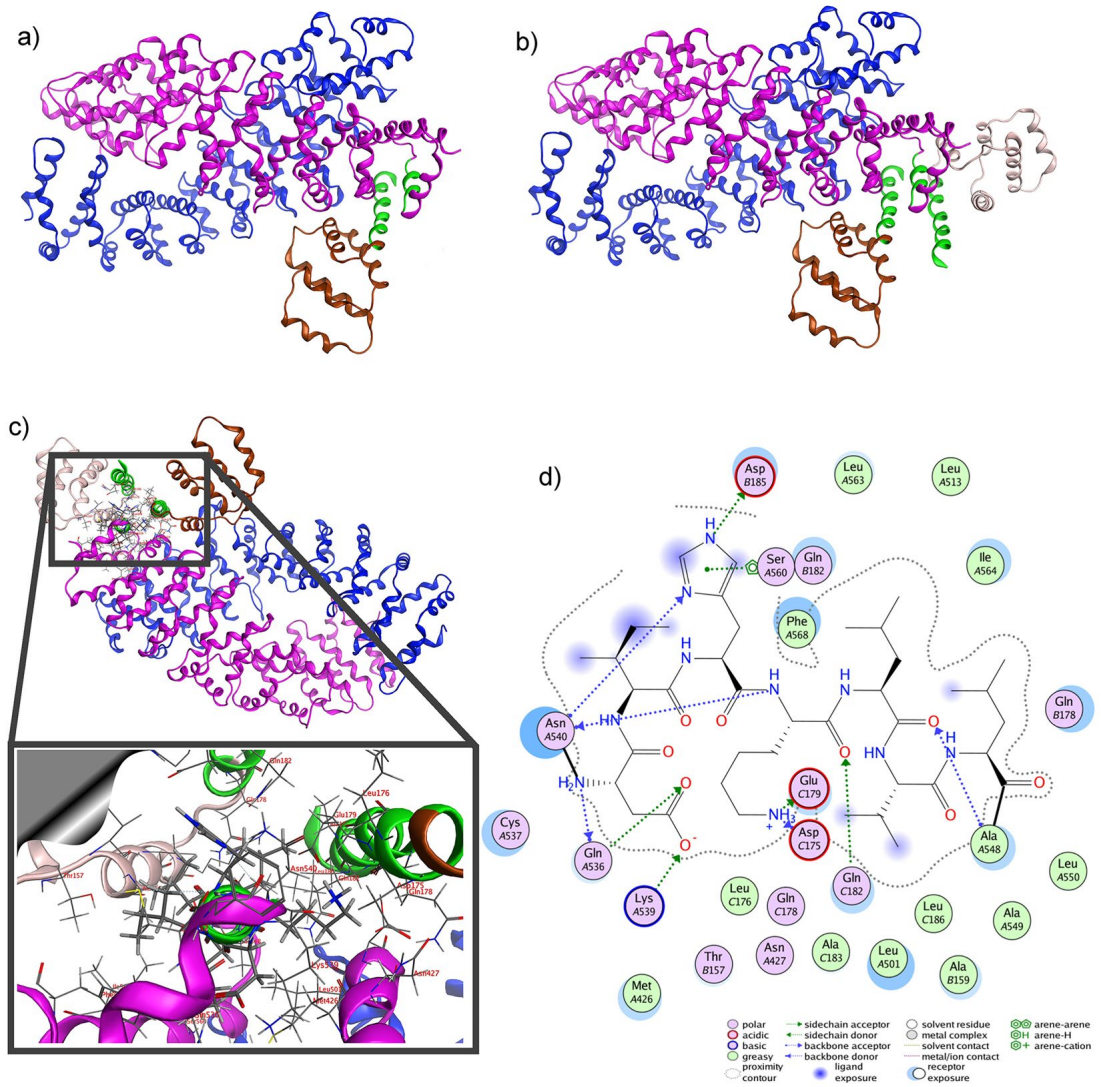
Molecular docking of LRRK2 ARM to FADD-DD. The model of dimeric LRRK2 ARM was used to dock the FADD DD crystal structure. The docking was performed iteratively placing each FADD DD structure onto the LRRK2 ARM model in sequence. (**a**) The FADD DD crystal structure (brown ribbon) was docked onto the LRRK2 dimer model (shown in blue and magenta ribbon, as before). (**b**) A second copy of the FADD DD crystal structure (silver ribbon) was docked to the molecular complex established in the previous step (**a**). The interaction was achieved via a network of antiparallel α-helical bundles (shown in green ribbon representation). (**c**) The network of molecular interactions between the specific residues involved is highlighted (inset). (**d**) The association between LRRK2 and FADD DD is stabilized via a mixture of ionic/hydrogen bonds, π-stacking and hydrophobic interactions. The amino acid notations are as follows: *A*, refers to the LRRK2 ARM sequence; and *B, C* refer to each FADD DD molecule.

The structure of the FADD DD was taken from the Fas/FADD crystal structure (PDB id: 3EZQ); and after energy minimization and MD structural optimization in a water cell, it was docked to the LRRK2 ARM dimer. The docking algorithm produced only one viable pose of FADD DD onto the LRRK2 ARM dimer (Fig. 5a). That pose was retained and the docking was repeated with a second FADD DD molecule; which markedly increased the stability of the complex. This reflects the tendency of FADD to form dimers mediated by interactions between corresponding DED’s^23^, as well as the fact that LRRK2 binds dimeric FADD DD considerably more strongly, when co-expressed in cells, than the monomeric death domain (^1^; and present work). In our model, the second molecule of FADD DD could only interact stably with the LRRK2 ARM dimer model if the first molecule of FADD was already docked (Fig. 5b), suggesting that the interaction of FADD slightly alters the local structural conformation of this region of LRRK2. Using this approach, 17 specific residues were identified within the N-terminal domain between amino acids Met532 and Lys547 as mediating the interaction with the FADD-DD (Fig. 5c,d).

**Figure 6:**
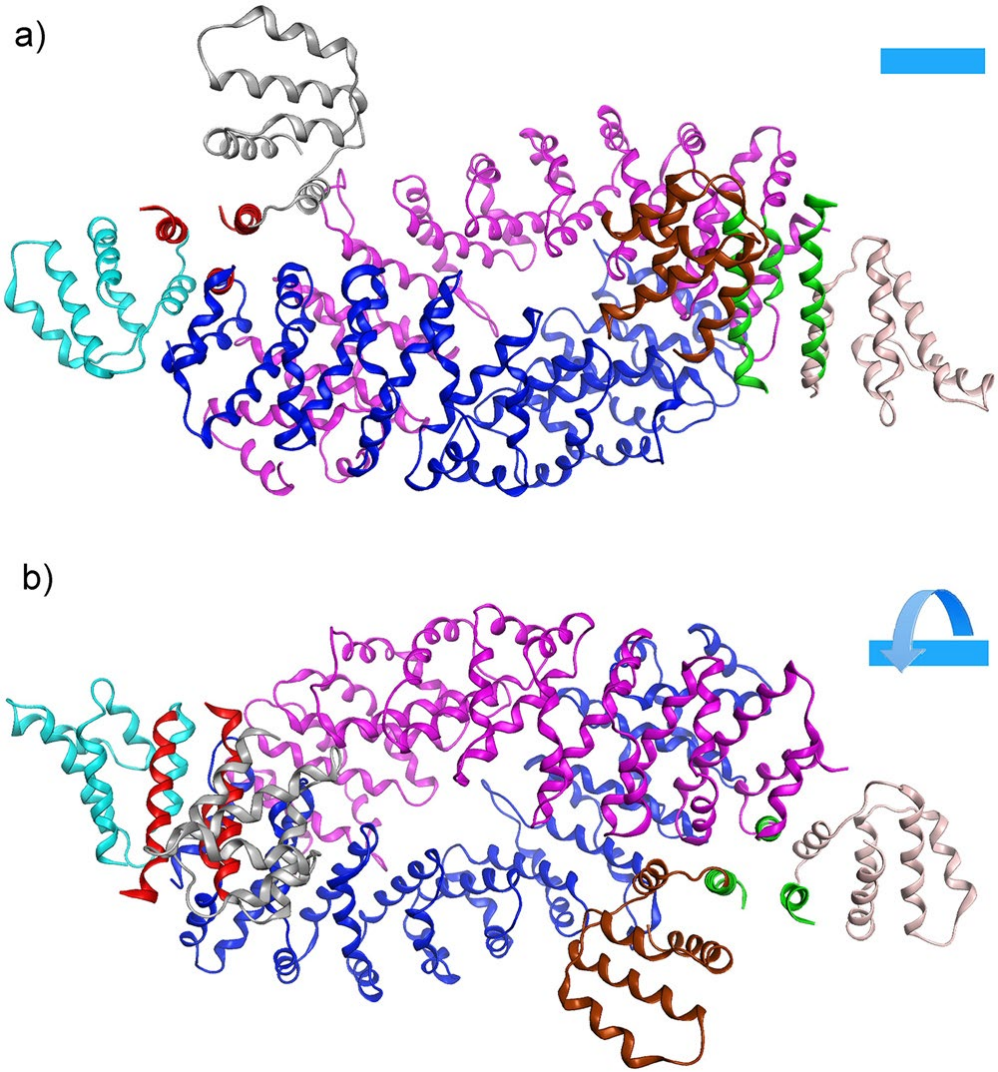
Molecular docking of the full LRRK2-FADD complex. (**a**) The dimeric model of LRRK2 ARM was used to dock two molecules of FADD DD on each end. By 180° rotation of the horizontal axis of the dimer interface (**b**), it is apparent that the axis of each group of triple-helix bundles is perpendicular to the other. Note that the established interacting α-helical bundles have been highlighted in red and green colored ribbon.

The docking of the FADD DD to the LRRK2 ARM model was influenced by the Fas/FADD interaction. In fact, the FADD DD’s docked to the LRRK2 model using the same α-helix by which it interacts with the Fas receptor, suggesting a common mode of interaction, and not unexpected given the similarity between the corresponding α-helical domains of Fas and LRRK2 (see Suppl Fig. 3). The resulting three α-helical bundles within the FADD DD create a strong hydrophobic core. The interaction is secured by a network of hydrogen bonds and π-stacking interactions (Fig. 5c,d). The LRRK2 interacting α-helix is supported within the 3D conformational space by intra molecular bonds, including from adjacent sites, within the LRRK2 ARM region (see Table 1). The two LRRK2 ARM models are predicted to interact via complimentary concaved surfaces that position the interacting sites in an optimal position to accommodate two FADD molecules on each side. Examination of the 3D arrangement of the LRRK2 ARM dimer indicated that the opposite protomer acts to support the interacting protomer, in order to maintain its original conformation (rotations; Fig. 6). This is illustrated by docking of the FADD-DD with only one LRRK2 monomer; such that the arch of the concave region of the LRRK2 ARM was increased and could not recover to its original conformation. Another crucial characteristic of the LRRK2 ARM model that is vital to its interaction with FADD is its neutral charge. The 3D model of the LRRK2 ARM revealed that the lack of highly charged regions, coupled with a hydrophobic inner concave surface, is critical for its interaction with FADD (Suppl Fig. 4). The binding efficacy of the LRRK2-FADD complex was evaluated by MD simulations that equilibrated the biological system in an aqueous environment of a periodic cell system (Suppl Fig. 5). The MD simulations of the dimeric LRRK2 ARM model with 2 FADD death domain molecules docked on each side equilibrated extremely quickly, thus confirming that the molecular system is stable (Suppl Fig. 6). Further, a series of molecular surfaces confirm the tight bonding of the helices via H-bonding, and due to the hydrophobic core that they established (Suppl Fig. 7).

**Table 1:** Summary of molecular interactions between LRRK2 and FADD.

Interacting atom	Residue	Residue number	Interaction	Distance	E (kcal/mol)
**Interactions between LRRK2 and the FADD DD monomers**					
NE2	ASP	185	H-donor	2.61	−1.8
NZ	ASP	175	H-donor	2.44	5.1
NZ	GLU	179	H-donor	2.26	99.8
O	GLN	182	H-acceptor	3.25	−2.0
NZ	GLU	179	ionic	2.26	−11.9
5-ring	GLN	182	pi-H	3.96	−0.6
**Intra LRRK2 interactions (with adjacent ARM repeats)**					
N	GLN	536	H-donor	2.63	−5.2
N	ASN	540	H-donor	3.17	−2.9
OD1	GLN	536	H-acceptor	2.49	3.7
OD2	LYS	539	H-acceptor	2.27	103.4
ND1	ASN	540	H-acceptor	3.59	−0.7
O	ALA	548	H-acceptor	2.91	−1.4
OD2	LYS	539	ionic	2.27	−11.7

We have recently found that a rare sequence variant in LRRK2, a substitution of glutamic acid for lysine at reside 544 (K544E), that has been reported in patients with familial PD^24^, induces apoptotic death of cultured primary neurons^7^. In this previous report, we showed that this variant displayed a slightly elevated interaction with FADD compared to WT-LRRK2. The Lys residue at position 544 lies within the identified FADD binding motif of the LRRK2 ARM repeat region. We investigated the structural effect of the K544E mutation using MD simulations (Suppl Fig. 8). The molecular systems in both cases reached equilibrium after 0,6–0,7 nanoseconds of simulation time. The glutamic acid in the place of the original lysine residue, at position 544, adds a predicted *de novo* hydrogen bond between the O-group of the glutamic acid and the side chain of the nearby Gln501. As a result, the FADD-interacting α-helix of this region of LRRK2 would be further stabilized by this new interaction, creating the conditions for FADD to bind more strongly.

### Targeting the LRRK2/FADD interaction is neuroprotective

Over-expression of a dimeric FADD death domain, by replacing the N-terminal DED with a leucine zipper motif^23^, confers protection against plasma membrane DR induced cell death. The mechanism of this protection is via displacement of endogenous FADD at the DR. Expression of the same dominant negative FADD-DD is neuroprotective in primary neurons expressing mutant LRRK2^1^; mediated by a strong interaction between dimeric FADD DD and LRRK2 that is markedly more stable than the interaction between LRRK2 and monomeric FADD DD^1^. We confirmed this using a full-length FADD construct containing a mutation that prevents dimerization (F25R^23^). As we have recently reported^1,7^, G2019S-LRRK2 binds WT FADD more strongly that WT LRRK2; however, both WT and mutant G2019S LRRK2 are unable to bind F25R-FADD (Suppl Fig. 9a); and like the prevention of the FADD-death receptor interaction by the dimeric FADD-DD^23^, the interaction between G2019S-LRRK2 and full length FADD is blocked by HA-tagged leucine zipper FADD-DD (Suppl Fig. 9a).

### Deletion of the predicted FADD binding domain in LRRK2-ARM prevents association with FADD and is neuroprotective

**Figure 7:**
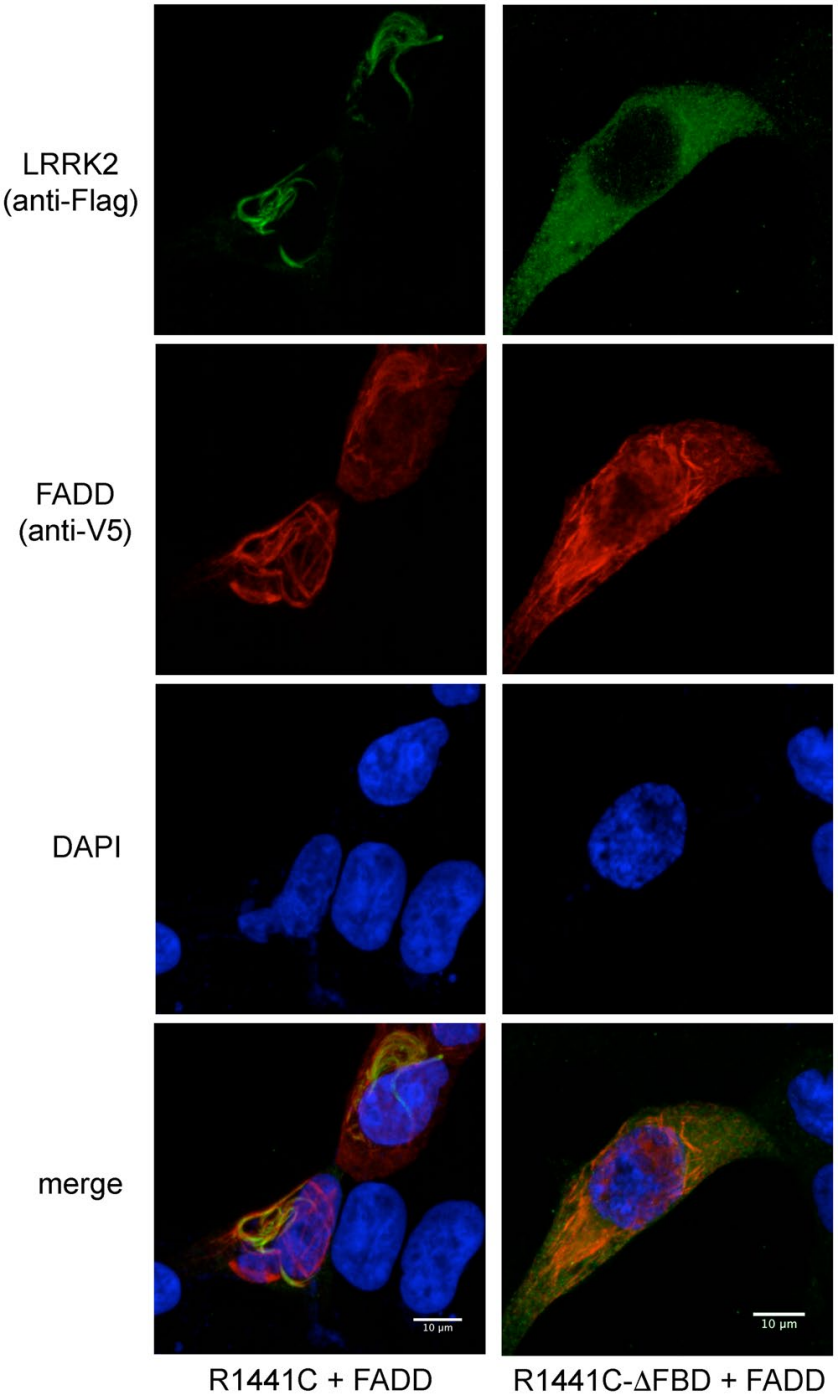
Deletion of predicted FADD binding domain in LRRK2 ARM prevents recruitment of LRRK2 to FADD complexes. Full-length mutant R1441C-LRRK2, or R1441C-LRRK2 lacking the predicted FADD binding domain (specifically, the residues within this motif with the highest identity, 538-547), was co-transfected with V5-FADD in HEK293T cells. Following 48 h of expression cells were fixed and immunostained for Flag (LRRK2) and V5 (FADD), together with DAPI. Over-expression of FADD leads to its localization to so-called Death Effector Filaments (DEFs) that subsequently recruits LRRK2. Deletion of the motif predicted to mediate binding to FADD (ΔFBD; FADD-binding domain) prevents this recruitment, leaving mutant LRRK2 diffusely localized in the cytoplasm.

**Figure 8:**
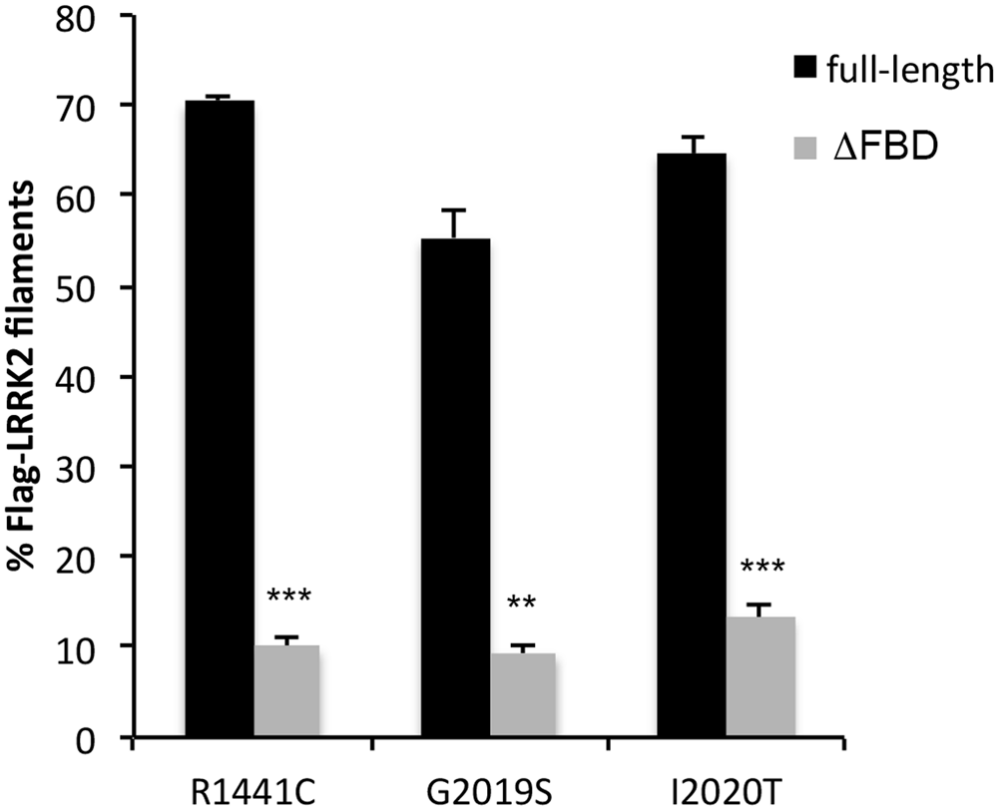
Quantification of LRRK2 recruitment to FADD DEFs. The percentage of LRRK2 filamentous structures co-localized with FADD DEFs was determined in a blinded fashion for each pair of full-length or deletion mutant LRRK2 constructs: R1441C, G2019S, and I2020T. **p < 0.01 compared to full-length mutant LRRK2; ***p, 0.001 compared to full-length mutant LRRK2. Each data point represents the mean +/− SEM from 3–4 independent transfections.

**Figure 9:**
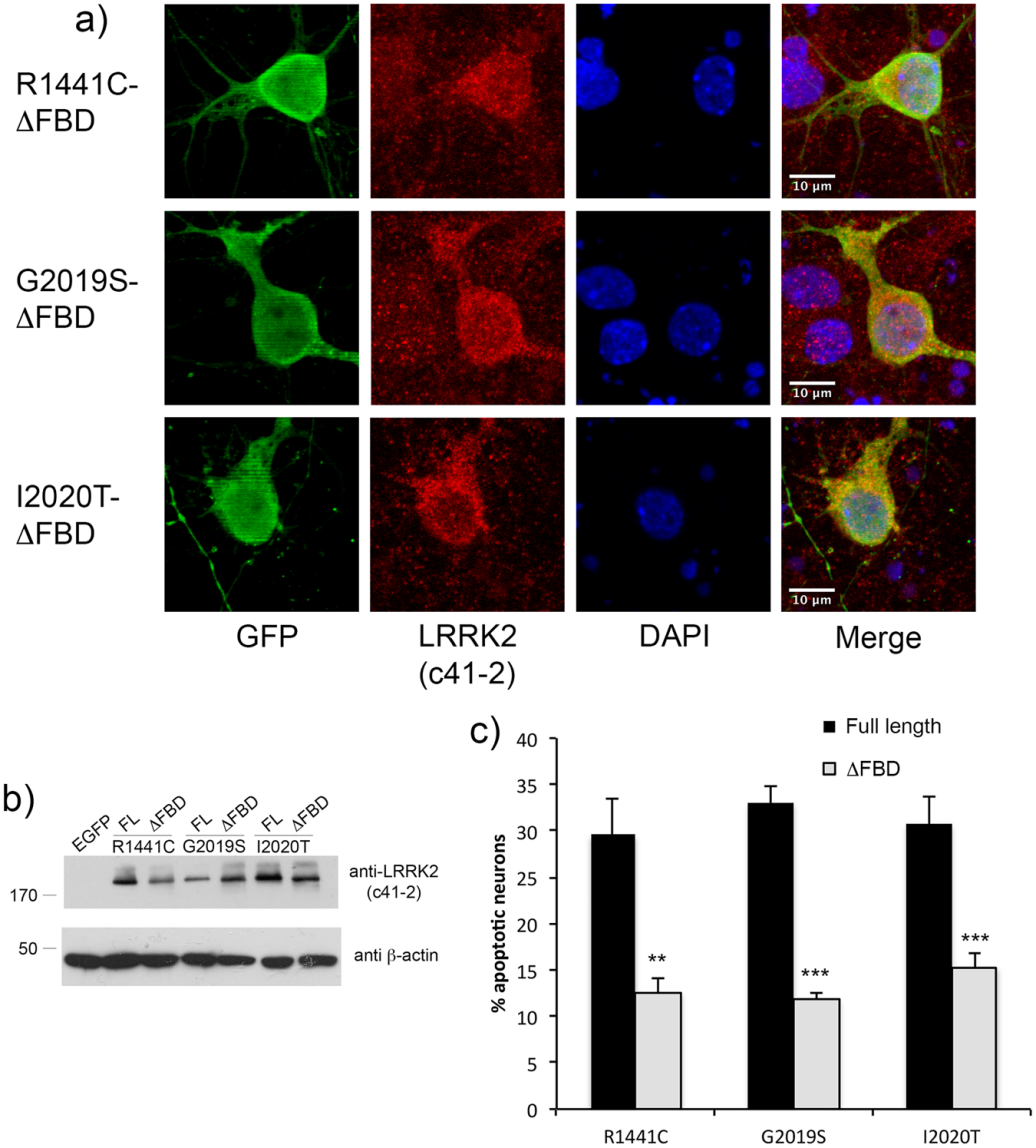
Deletion of predicted FADD binding domain in LRRK2 ARM region is neuroprotective. Primary rat embryonic cortical neurons were transiently transfected with full-length mutant, or its corresponding deletion pair (ΔFBD), together with a construct encoding an EGFP reporter at a ratio of 4:1. Following 72 h of expression, neurons were fixed and processed for anti-LRRK2/EGFP immunofluorescence with DAPI nuclear stain (**a**). Please see Fig. 2 and Supplementary Fig. 11 for representative images of full-length mutant LRRK2. Panel (b) shows the relative expression levels of full-length or deletion constructs. HEK293T cells were transiently transfected with full-length mutant LRRK2, or mutant LRRK2 (ΔFBD) lacking the FADD binding domain. Cell extracts were separated by SDS-PAGE and the membranes probed with anti-LRRK2 (c41-2) and β-actin, as a loading control. In (**c**), the percentage of EGFP-positive neurons displaying apoptotic nuclear morphology was determined by a rater blinded to the experimental conditions. **p < 0.01 compared to full-length mutant LRRK2; ***p < 0.001 compared to full-length mutant LRRK2. Each data point represents the mean +/− SEM from 3-4 independent transfections. Cultures were repeated at least 3 times with similar differences.

We created a series of deletion constructs in which the key predicted residues within the LRRK2 ARM region are deleted (amino acids 538-547). These deletion constructs were created on the backbone of WT, R1441C-, G2019S-, and I2020T-LRRK2. We co-expressed each full-length or its matching deletion construct together with V5-FADD in HEK293T cells, and determined by co-immunoprecipitation as well as by the FADD “death effector filament” (DEF)-recruitment assay, whether the interaction with FADD was affected. In HEK293T cells, the immunoprecipitation of Flag-LRRK2 was less efficient in the presence of this deletion, making quantification of the loss of FADD co-precipitation difficult (not shown). However, as can be seen in Fig. 7 at the single cell level, deletion of the predicted residues in the LRRK2 ARM region prevented the recruitment of LRRK2 to FADD-positive DEF’s (quantified in Fig. 8), indicating a reduction in the interaction between LRRK2 and FADD. Shown in Fig. 7 are representative images from full-length R1441C-LRRK2 or it’s deletion counterpart, co-transfected with FADD. Representative images of G2019S- or I2020T-LRRK2 full-length and deletion mutants are shown in Suppl Fig. 10. Next, we transiently expressed the full-length, or matching deletion, constructs for each of the three pathogenic mutant forms of LRRK2 in primary rat cortical neurons. After a period of 72 h, the neurons were fixed and processed for LRRK2 expression and nuclear morphology. Immunofluorescent staining of full-length or LRRK2 with the deletion in the ARM region revealed similar levels of expression in primary cortical neurons (see Fig. 9a for anti-LRRK2 staining; see Fig. 2, and Suppl Fig. 11 for representative images of neurons expressing full-length LRRK2); and Western immunoblotting confirmed this in HEK293T cells (Fig. 9b). Counts of apoptotic nuclei revealed that deletion of the predicted FADD-interacting domain in the LRRK2 ARM region reduced the extent neuronal apoptotic death induced by mutant LRRK2 (Fig. 9c).

**Figure 10:**
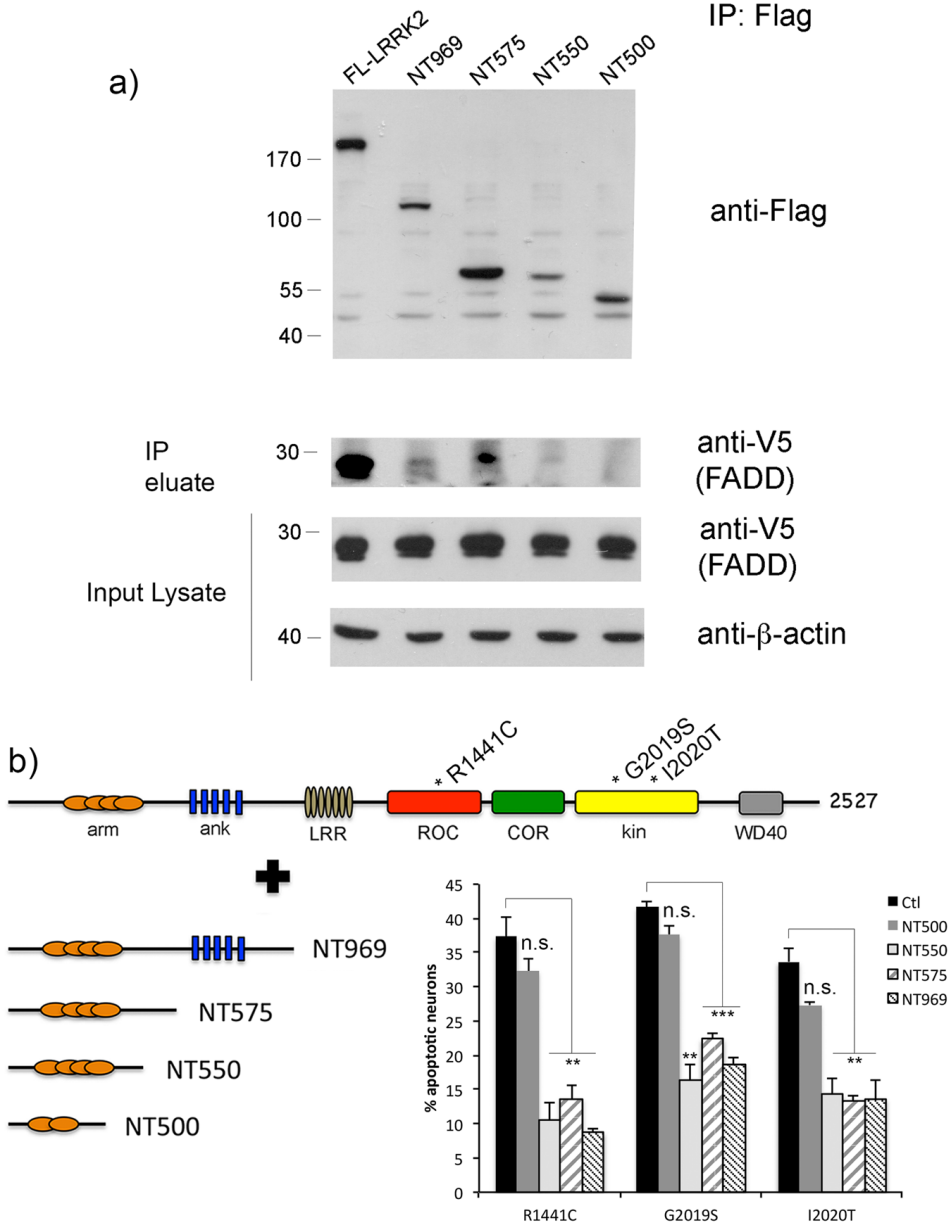
Fragments of LRRK2 containing FADD binding motif are neuroprotective. (**a**) HEK293T cells were co-transfected with V5-FADD and Flag-tagged full-length WT or mutant LRRK2 (I2020T); or fragments of the N-terminal domain of LRRK2 comprised of amino acids 1-500 (NT500), 1-550 (NT550), 1-575 (NT575), or 1-969 (NT969). Cell extracts were subjected to anti-Flag immunoprecipitation and the eluates were probed for the presence of co-precipitating FADD. While a fragment of the LRRK2 N-terminal domain containing amino acids 1-500 (NT500) was unable to bind FADD, increasing the length to 550 or 575 amino acids (NT500, NT575) restored the interaction with FADD, suggesting that the FADD binding motif is located within residues 500-575 of the LRRK2 N-terminal domain. (**b**) Primary cortical neurons were transiently co-transfected with full-length WT or R1441C, G2019S, or I2020T mutant LRRK2 together with the different N-terminal fragments of LRRK2 depicted in the schematic. Fixed neurons were then assessed for the presence of apoptotic nuclear features. Co-expression of the fragments of LRRK2 that interact with FADD blocked the apoptotic death of cortical neurons induced by full-length G2019S mutant LRRK2. **p < 0.01 compared to neurons transfected with R1441C or I2020T LRRK2 and pcDNA control plasmid; ***p < 0.001 compared to neurons transfected with G2019S-LRRK2 and pcDNA control plasmid. Each data point represents the mean +/− SEM from 3–4 independent transfections. Cultures were repeated at least 3 times with similar differences.

We next created a set of smaller fragments within the N-terminal region of LRRK2. Flag-tagged fragments containing the first 500 amino acids (NT500), the first 550 amino acids (NT550), or the first 575 amino acids (NT575), were tested in parallel with the full N-terminal domain (NT969). The region between residues 500 and 969 was not stable when over-expressed in HEK293T cells, however since our *in silico* findings led us to a motif within the first 550 amino acids of the protein, we focused the biochemical studies on these fragments. We found no significant interaction between the NT500 fragment and FADD (Fig. 10a). In contrast, when the NT575 fragment was co-expressed, the ability to co-precipitate with FADD was restored, as well as when a shorter fragment comprised of residues 1-550 (NT550) was co-expressed with FADD. Collectively, the *in silico* modeling coupled with the biochemical findings suggest that the FADD-interaction motif is located within the 500 to 575 amino acid region of the N-terminal domain of LRRK2.

**Figure 11:**
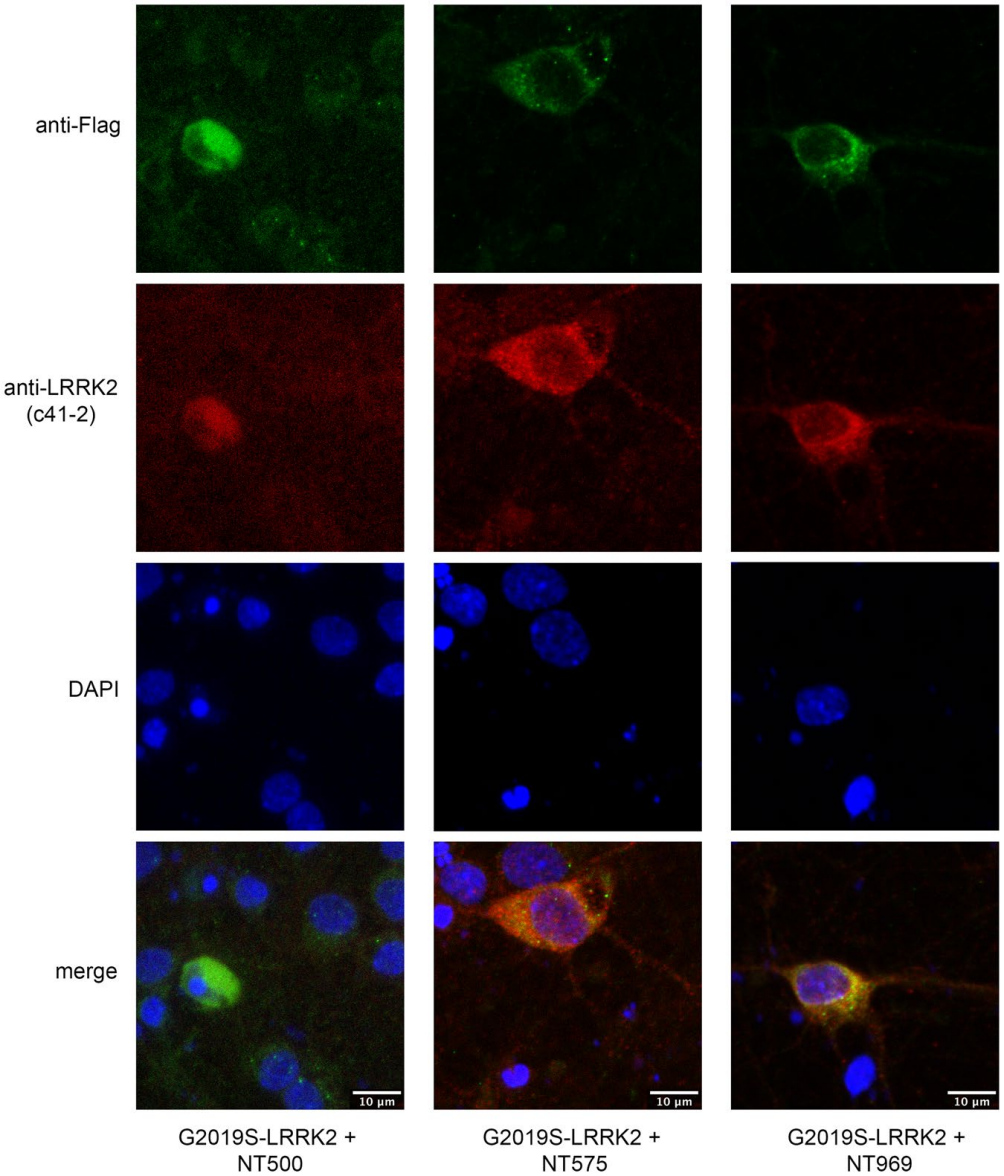
Fragments of LRRK2 ARM region are neuroprotective. Primary cortical neurons were transiently co-transfected with full-length untagged WT or G2019S mutant LRRK2 together with different Flag-tagged N-terminal fragments of LRRK2 depicted in the schematic shown in Fig. 10b. Fixed neurons were then processed for anti-Flag immunostaining to label the LRRK2 ARM fragment (NT500, NT575, or NT969), together with anti-LRRK2 (clone c41-2) to label full-length LRRK2. A representative image showing neurons expressing G2019S-LRRK2 together with the different ARM fragments is presented. Scale bar is 10 μm.

We used the same primary rat cortical neuron model to determine whether prevention of the interaction with (endogenous) neuronal FADD blocks neuronal death induced by full-length mutant LRRK2. As would be predicted, prevention of the interaction between over-expressed mutant LRRK2 and endogenous neuronal FADD by co-expression of the entire N-terminal domain or the smaller N-terminal sub-fragments is neuroprotective (Fig. 10b). Representative images of neurons co-expressing un-tagged mutant LRRK2 together with Flag-tagged N-terminal (ARM) fragments of LRRK2 are shown in Fig. 11. Additional images of mutant LRRK2-expressing neurons co-transfected with NT500 or NT575 are shown in Suppl Fig. 11. Additionally, as expected, we also observed that neuronal death induced by either R1441C or I2020T mutant LRRK2 was also similarly disrupted by the dominant negative-mediated disruption of FADD binding. This protection is associated with the ability of the N-terminal fragment to co-immunoprecipitate with FADD. The smaller N-terminal fragment consisting of the first 500 amino acids is unable to co-immunoprecipitate with FADD, and thus does not block neuronal death induced by full-length mutant LRRK2 (Fig. 10b); whereas the longer N-terminal fragments, NT550, NT575, and NT969 confer neuroprotection.

## Discussion

In this study, we provide an in depth characterization of the death signaling steps that are triggered in neurons following expression of mutant forms of the ROCO/RIP kinase LRRK2. While it is known that expression of mutant forms of this protein activate late stage effector caspases such as caspase-3^8,9^, the mechanism leading to this activation was poorly understood. We now show that the activation of FADD and caspase-8 pathways recruit intrinsic mitochondrial apoptotic proteins such as Bid, and its co-factor Bax. We have mapped the LRRK2-FADD DD interaction domain to a short motif within the N-terminal armadillo repeat using 3D molecular modeling. Importantly, in primary neurons, the ability to disrupt the interaction between mutant LRRK2 and FADD confers neuroprotective properties to fragments of the LRRK2 ARM repeat that contain the FADD interacting motif.

FADD is comprised of two functional domains, an N-terminal death effector domain (DED) that mediates the interaction of FADD with procaspase-8 and 10 ^25^, and a C-terminal DD that binds the plasma membrane receptor, as well as LRRK2^1^. FADD also appears to mediate the interaction between LRRK2 and caspase-8, as in the absence of FADD, the interaction between LRRK2 and caspase-8 is almost absent^1^. The F25 residue (together with a Lys at position 33) has previously been implicated, in addition to FADD self-association^23,26^, but also in the interaction between FADD and procaspase-8^27^ or cFLIP^28^; and expression of F25R mutant FADD suppresses the response to FasL^26^. Mutation of this residue abolishes the ability of FADD to be recruited to the plasma membrane DRs^23^. Replacement of the N-terminal DED with an artificial dimerization motif (leucine zipper) successfully competes against endogenous full-length FADD for binding to plasma membrane DR upon activation^23^. Similarly, we have previously found that this truncated dimeric DD fragment of FADD binds very strongly to LRRK2^1^, and blocks neuronal death induced by mutant LRRK2. In the present work, we show that over-expression of lz-DD FADD also blocks the interaction of G2019S-LRRK2 with full-length FADD. Along these lines, and similar to the loss of DR binding, the F25R FADD mutant is unable to bind WT or mutant G2019S LRRK2. These findings indicate that LRRK2 preferentially binds dimeric FADD. The neuroprotection against mutant LRRK2 expression provided by the dimeric FADD-DD fragment is dependent on its ability to prevent LRRK2 from interacting with full-length FADD. It is possible that a similar mechanism underlies the neuroprotection provided by the LRRK2 ARM fragment, which we demonstrate here binds strongly to full-length FADD.

The homology model of the LRRK2 ARM dimer revealed a supercoiled structure of ARM repeats that establish a tube-like formation similar to that of coiled-coil structures (see Fig. 4). This overall organization is consistent with the recently described LRRK2 model^21^. The compact 3D structure of the LRRK2 helices in this region is permitted by the absence of bulky residues that would disrupt the folding of this stretch of LRRK2 ARM. There are only 5 tryptophan and 7 tyrosine residues, which is 0.86% and 1.21%, respectively, of this entire region; in contrast to 84 leucine residues (14.6%). This permits the establishment of the characteristic ARM configuration underlying its protein-protein interaction role. Structurally, it is a critical conformation that enables the arched LRRK2 ARM domain to be able to tolerate the impact of the iterative binding of the four FADD molecules.

The C-terminal region of the LRRK2 ARM mediates the physical interaction with the FADD DD, but the role of the adjacent more flexible N-terminal region of the counterpart monomer is pivotal in terms of shape and size complementarity of the protein – protein interaction, as this region absorbs the slight conformational change upon binding of the two FADD molecules (see Fig. 5b). The docking results confirmed that the interacting α-helix of LRRK2 is a short motif within residues 532 and 547, consistent with our biochemical observations that fragments of the first 500 amino acids of the N-terminal domain failed to bind FADD, whereas longer fragments of 550 or more amino acids retained the interaction. The Fas/FADD X-ray structure was used to predict the precise location and positioning of the two α-helices within the LRRK2 ARM repeats. The identified motif within the LRRK2 ARM region shares significant sequence similarity to the region of the Fas receptor that binds FADD (Figure Sup 3). The structure of the FADD interacting motif within LRRK2 is α-helical, as is Fas. Therefore, the LRRK2 ARM model was superposed on the critical region within the Fas crystal structure during the docking process in order to optimize the docking with the FADD DD.

Although we utilized the ARM-containing protein Rch1/importin-alpha1 (PBD id: 3WPT) as a template for the modeling of the LRRK2 ARM, there are also some intersecting functional considerations for these two proteins. Importin alpha-1 is involved in the nuclear import of stress-induced transcription factors such as NFAT and Nrf2^29^. In the case of NFAT, its transport to the nucleus is negatively regulated by LRRK2^30^. Moreover, LRRK2 may play a role in the import of Nrf2, via its interaction with p62, as recently reported^31^. Likewise, this cellular activity may also come into play concerning the interaction between LRRK2 and FADD. Since the wild type form of LRRK2 readily binds FADD, the key question remains concerning the mechanism of action of FADD-dependent death signaling when mutant LRRK2 is present. The retention of FADD within the nucleus suppresses its death-inducing activity in T cells^32^. The baseline interaction with WT LRRK2 may direct a portion of FADD molecules to be shuttled to the nucleus via a conventional nuclear import mechanism (as LRRK2 is not present in the nucleus or nuclear membrane). One could speculate that this regulatory function is disrupted by mutant LRRK2, akin to a loss of a protective function.

Early work by multiple groups has shown that the kinase activity of LRRK2 is necessary for its induction of cell death^4,5,33^; yet paradoxically, only the G2019S mutant shows a robust increase in kinase activity^28^. However, absent physiological phospho-substrates linked to cell death pathways, coupled with emerging evidence that the kinase-dependency may be linked to the regulation of LRRK2 levels rather than the phosphorylation of specific substrates^2,34^, this issue remains unresolved. The recent identification of broad increases in phosphorylation of certain Rab GTPase family members by multiple LRRK2 variants^35^ may provide a possible mechanistic basis for the universal kinase-dependent induction of neuronal death, or at the very least suggests that other physiological death-related substrates may yet be identified that are hyper-phosphorylated by mutant LRRK2 regardless of the specific mutation. While there is no evidence to date that LRRK2 directly phosphorylates FADD, inhibition of kinase activity alters its interaction with FADD^1^, possibly due to altered levels or cellular distribution of LRRK2.

The finding that inhibition of Bid and Bax signaling blocks neuronal death induced by mutant LRRK2 is significant in that it establishes a link between early activation of FADD and caspase-8 signaling and late stage effector caspase-3. One of the targets of caspase-8 activity is Bid, and translocation of the truncated form of Bid to mitochondria, where it acts as a co-factor with Bax^36,37^, is prevented by the BI-6C9 peptide inhibitor^16^. Previous work had implicated mitochondrial involvement in mutant LRRK2-induced death, including the phosphorylation of Bcl-2^38^, which awaits further validation; the interaction of LRRK2 with mitochondrial permeability protein regulators^39^, and the disruption of apoptosome formation^9^. The present findings define the mechanism of activation of mitochondrial events in the control of the death pathway activated in neurons expressing mutant LRRK2.

**Figure 12:**
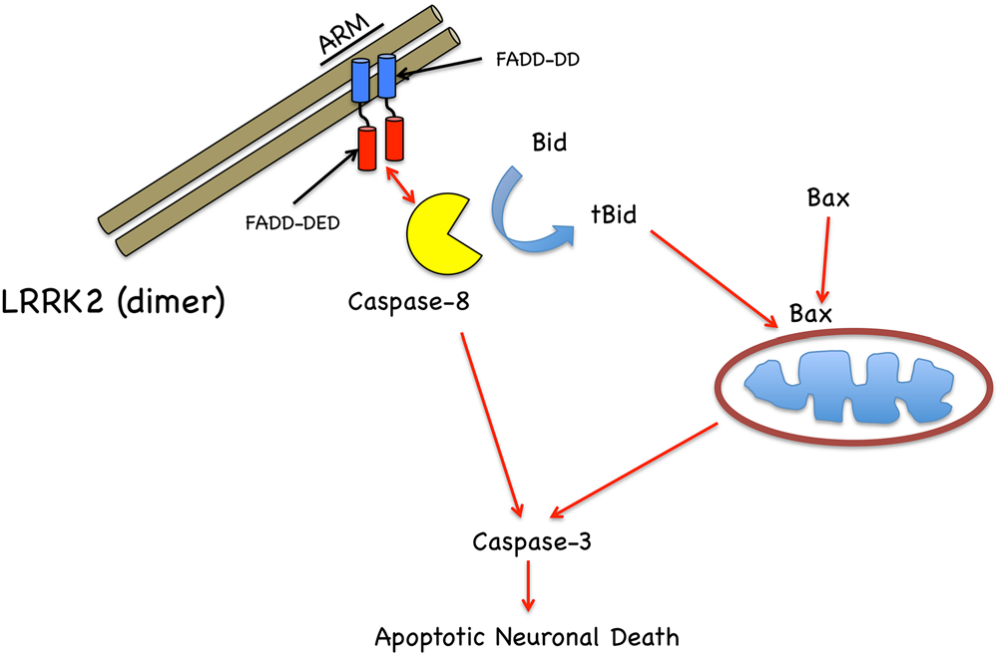
Proposed model of FADD/Bax-dependent neuronal death induced by mutant LRRK2. Dimeric mutant LRRK2, binding FADD through its ARM repeat region located within the N-terminal domain, recruits and activates caspase-8. We show here that the intrinsic mitochondrial apoptotic machinery is in turn activated through the pro-apoptotic Bcl-2 proteins, Bid and Bax.

The related kinase, LRRK1 lacks the N-terminal ARM repeats that are present in LRRK2. We show now that FADD binds LRRK2 selectively within this region, whereas LRRK1 fails to co-precipitate with FADD. Interstingly, mutations in LRRK1 equivalent to corresponding residues in LRRK2 fail to induce neuronal death^40^. The absence of a corresponding protein-protein interaction region within LRRK1, that potentially alters the full profile of interacting proteins, including FADD, may underlie the lack of neurotoxicity triggered by mutations in LRRK1. Taken together, a potential mechanism of action is emerging in which mutant LRRK2 behaves in a similar manner to ligand-bound plasma membrane DRs in terms of activating FADD. One of the key questions remaining is to better understand the transition between the baseline interaction between FADD and WT LRRK2, and the toxic interaction with mutant LRRK2; whether this is strictly regulated by an increase in this interaction, or some other signaling events. We use two complimentary approaches to exogenously block the interaction of mutant LRRK2 with FADD: over-expression of a fragment of LRRK2 containing the FADD binding motif, and expression of a dimeric FADD fragment that blocks mutant LRRK2 from binding full-length endogenous FADD; and each of these approaches result in increased neuronal survival. Further, the fact that deletion of the short motif predicted *in silico* to mediate the interaction between LRRK2 and FADD is neuroprotective bolsters this model of activation. We find that LRRK2 lacking these residues in the ARM repeat region, fails to localize to over-expressed FADD DEFs, suggesting that the toxic signaling pathway may be initiated at the endogenous equivalent of FADD DEF complexes. A schematic of the proposed model of LRRK2-FADD-Bax dependent neuronal death signaling is depicted in Fig. 12. Downstream of the activation of FADD, we now describe the recruitment of Bid/Bax-dependent apoptotic machinery in neuronal death caused by mutant LRRK2. The canonical link between these two pathways is the caspase-8 mediated cleavage of Bid; and we find that inhibition of cleaved Bid is neuroprotective in a cellular model of mutant LRRK2 induced neurotoxicity. Our genetic as well as pharmacological evidence that inhibition of Bax signaling is also neuroprotective in this model is consistent with evidence that tBid and Bax function as co-factors upon translocation to mitochondria to propagate the cellular death signal^41^. Together, these findings provide a strong rationale for targeting other upstream signaling points as a potential therapeutic strategy to be used alongside, or as an alternative to, inhibition of LRRK2 kinase activity.

## Materials and Methods

### Plasmids

Human LRRK2 cDNA with an N-terminal Flag epitope tag was used as described^7^. Full-length V5-tagged WT or F25R mutant FADD, as well as HA-tagged FADD dominant negative (lz-DD) were used as previously described^1^. Constructs expressing Flag-tagged LRRK2 domains were cloned from the full length WT cDNA using the domain boundaries indicated in Fig. 3A. Similarly, shorter fragments of the N-terminal domain (1-969) were cloned using this domain as a template. All LRRK2 fragments were fully sequenced prior to use. Deletion constructs consisting of full length WT or mutant LRRK2 lacking the predicted FADD binding domain in the ARM region (the residues predicted to interact most strongly, amino acids 538-547) were created using the Quikchange II site-directed mutagenesis kit (Agilent Technologies, CA, USA). Deletion of the candidate motif was confirmed by sequencing. A plasmid containing human WT myc-LRRK1 cDNA was a generous gift of Dr. Mark Cookson (NIA/NIH). The LRRK1 cDNA was PCR-amplified with primers encoding an N-terminal Flag epitope tag, and the product was cloned into the pcDNA3.1 expression vector and fully re-sequenced.

### Cell Lines and Primary Neuronal Cultures

HEK293T cells (ATCC) were grown in DMEM medium (Sigma) supplemented with 10% FBS and penicillin/streptomycin. Primary embryonic E17 rat cortical neurons were established as described^42^. Dissociated neurons were then counted and plated on poly-d-lysine coated glass coverslips in Neurobasal medium containing B-27 serum-free supplements (Invitrogen) and penicillin/streptomycin. Following four days in culture, neurons were transfected with Lipofectamine 2000 (Invitrogen) according to the manufacturer’s instructions. In co-expression experiments, full-length LRRK2 expression constructs were transfected with FADD or LRRK2 dominant negative constructs, as indicated, at a ratio of 4:1. The use of animals in this study for the preparation of primary embryonic cortical neurons was reviewed and approved by the Ethical Committee for Use of Laboratory Animals in BRFAA. Bax+/+ or −/− cortical neurons were prepared from E16 mouse embryos resulting from heterozygous breeding. Neurons from each individual embryo were plated separately and genotyping was performed by PCR from tail DNA. The primers used were as follows: 5′-GTTGACCAGAGTGGCGTAGG-3′; 5′-CCGCTTCCATTGCTCAGCGG-3′; and 5′-GAGCTGATCAGAACCATCATG-3′^11^. Survival of primary cortical neurons expressing LRRK2 was determined as follows: fixed neurons were immunostained for GFP together with active caspase-3 to label apoptotic neurons, which were defined as those having condensed fragmented chromatin comprised of two or more apoptotic bodies in combination with active caspase-3 labeling. More than 100 neurons per coverslip were assessed in triplicate in a blinded fashion, from three to four independent cultures. All transgenic mice used in this study were treated according to NIH guidelines for Care and Use of Laboratory Animals, and with the approval of Columbia University’s Institutional Animal Care and Use Committee.

### Antibodies

Rabbit anti-GFP (Abcam) was used to detect the EGFP reporter co-transfected with Flag-LRRK2 in primary neurons. We used mouse anti-Flag (clone M2; Sigma); rabbit anti-V5; rabbit anti-LRRK2 (UDD3; Abcam) or mouse anti-LRRK2 (N241A/34; Antibodies Incorporated); and anti-Flag agarose beads were from Sigma or Biotool. Mouse anti-V5 was from Invitrogen/Thermo Scientific. Rabbit polyclonal anti-active caspase-3 was obtained from R&D Systems.

### Immunofluorescent Labeling and Confocal Imaging

At the indicated times following transfection, cells grown on glass coverslips were fixed in 4% paraformaldehyde for 20 min at 4 °C, washed in PBS and blocked in PBS containing 0.25% Triton X-100 and 10% normal donkey serum. Coverslips were then incubated overnight at 4 °C with primary antibodies diluted in PBS containing 0.25% Triton X-100 and 1% normal donkey serum. The following day, the coverslips were washed with PBS and incubated with secondary antibodies conjugated with Alexa 488 or Alexa 594 in the same diluent as for the primary antibodies for 1hr at room temperature, followed by staining with DAPI. Confocal images were obtained on a Lecia SP5 two-photon microscope.

### Immunoprecipitation and Western Immunoblotting

HEK293T cells transfected with the various expression constructs were lysed by Dounce homogenization in a buffer containing: 20 mM HEPES, pH 7.4; 150 mM NaCl; 0.1% NP-40; 2 mM EGTA; 2 mM MgCl_2_; 10% glycerol; pH 7.2; phosphatase inhibitor cocktail and protease inhibitors (Roche). Following centrifugation to remove insoluble material, the lysate was pre-cleared with protein-G agarose beads for 1 h. The cleared lysate was then incubated with primary antibody overnight at 4 °C followed by addition of protein-G beads, or directly with anti-Flag resin (Sigma), and allowed to incubate under constant rotation at 4 °C. The following day, the beads were washed in lysis buffer five times, followed by boiling in 2X Laemmli/SDS sample buffer for 5 min to release the immunocomplexes. Samples were then separated by SDS-PAGE and blotted membranes blocked with 5% non-fat milk and incubated with primary antibodies (as indicated) overnight at 4 °C. The following day, the membranes were further washed and incubated with HRP-conjugated secondary antibodies (Pierce) for 1 h at room temperature.

### FADD recruitment assay

Over-expression of several extrinsic pathway proteins, including FADD, leads to the formation of cytoplasmic Death Effector Filaments (DEF’s), produced via oligomerization^43,44^. We have previously observed a selective recruitment of LRRK2 only to such DEF’s formed by FADD (W.T. Dauer, personal communication). To monitor the interaction of FADD and WT or mutant LRRK2 in cells, we utilized this approach. HEK293T cells on glass coverslips were transiently transfected with V5-FADD and Flag-tagged LRRK2, fixed following 48 h after transfection, and processed for V5 and Flag immunofluorescence. The percentage of Flag-positive cells in which the Flag immunofluorescence signal assumed a filamentous distribution within the cytoplasm was determined.

### Structural Modeling of LRRK2 N-terminal region

#### Molecular modeling

All calculations were performed on a quad SLI nVIDIA GTX based cluster featuring 2304 CUDA cores in each of the four GPUs. The cluster is operated via a 64 bit Unix based operating system and multi-core and multi-threading management has been achieved via in-house programmed routines specifically customized for the computational needs of this study.

#### Homology modeling

All homology models were constructed using MOE version 2014. Homology modeling of LRRK2 N-terminal region was based on the crystal structure of human Importin-alpha1/rch1 (PDB id: 3WPT), which shares 30% identity to the armadillo repeat region of LRRK2. The first step in the construction of the LRRK2 model was a sequence alignment against the selected 3WPT template. The quality of this model was examined by a repertoire of built-in modules within MOE (including protein packing, geometry and free energy perturbation scoring algorithms). The final 3D model was subjected to energy minimization using the Amber94 force field as implemented in MOE. The refinement of the constructed homology model was achieved via energy minimization. A total of 10000 steepest descent iterations were followed by a conjugated gradient calculation, up to a gradient of convergence equal to 0.0001 kcal/mole Å^−1^.

#### Molecular Docking

The module Glide 1 of the Schrödinger suite was used for the molecular docking experiments. The molecular building and coordinate preparation of the system was achieved using Maestro program2 prior to docking. The crystal structure of FADD DD from the FADD DD/Fas complex crystal structure (PDB id: 3EZQ^45^); was docked onto the 3D model of LRRK2 in dimeric form. The Protein Preparation Wizard3 module of Maestro was used to prepare the structures and models to be docked. All crystallographic water molecules were removed from the modeled proteins prior to docking. Hydrogen atoms and partial charges were added to the all proteins and the initial structures were energetically minimized using the CHARMM force field. Next, the molecular system was re-built with the LRRK2 dimer and the docked monomer of FADD DD from the previous round to dock a second molecule of the FADD DD. The docking experiment was repeated on the other LRRK2 protomer resulting in four FADD DD molecules in total docked on the LRRK2 dimer.

#### Molecular dynamics (MD) simulations

MD simulations were performed using MOE version 2014 and its built-in Molecular Dynamics module using an NVT ensemble. Initially the molecular system was solvated in a cubic periodic box. The periodic box was explicitly filled with SPC (single point charge) water molecules. The models were refined by adding all hydrogen atoms and performing energy minimizations using the CHARMM force field. The time step for all calculations was set to 2 fs and the temperature at 300 K. The model was first equilibrated for 100 ps keeping the whole protein fixed to allow the water molecules to relax. A subsequent 100 ps of equilibration with the protein backbone fixed was carried out. After the equilibration phase, we obtained 10 ns MD trajectory for the LRRK2 model. Finally, a conjugate gradient energy minimization of the full protein was performed until the root mean-square (rms) gradient energy was lower than 0.0001 kcal/mol Å^−1^.

### Statistical Analyses

For statistical comparisons, we employed GraphPad Prism, utilizing the one-way ANOVA test, with Tukey’s HSD test for post-hoc pairwise comparisons. For each Figure, the *p* value is indicated in the associated Legend. The data are presented as the mean +/− standard error of the mean. The number of biological and experimental replications is provided in the associated Figure Legend where appropriate.

## Electronic supplementary material

Supplementary Data

## References

[1] Ho CC, Rideout HJ, Ribe E, Troy CM, Dauer WT (2009). The Parkinson disease protein leucine-rich repeat kinase 2 transduces death signals via Fas-associated protein with death domain and caspase-8 in a cellular model of neurodegeneration. J Neurosci.

[2] Skibinski G, Nakamura K, Cookson MR, Finkbeiner S (2014). Mutant LRRK2 toxicity in neurons depends on LRRK2 levels and synuclein but not kinase activity or inclusion bodies. J Neurosci.

[3] Smith WW (2005). Leucine-rich repeat kinase 2 (LRRK2) interacts with parkin, and mutant LRRK2 induces neuronal degeneration. Proc Natl Acad Sci USA.

[4] West AB (2007). Parkinson’s disease-associated mutations in LRRK2 link enhanced GTP-binding and kinase activities to neuronal toxicity. Hum Mol Genet.

[5] Greggio E (2006). Kinase activity is required for the toxic effects of mutant LRRK2/dardarin. Neurobiology of disease.

[6] Kett LR (2012). LRRK2 Parkinson disease mutations enhance its microtubule association. Hum Mol Genet.

[7] Melachroinou K (2016). Activation of FADD-Dependent Neuronal Death Pathways as a Predictor of Pathogenicity for LRRK2 Mutations. PLoS One.

[8] Chen CY (2012). (G2019S) LRRK2 activates MKK4-JNK pathway and causes degeneration of SN dopaminergic neurons in a transgenic mouse model of PD. Cell Death Differ.

[9] Iaccarino C (2007). Apoptotic mechanisms in mutant LRRK2-mediated cell death. Hum Mol Genet.

[10] Guicciardi ME, Gores GJ (2009). Life and death by death receptors. FASEB journal: official publication of the Federation of American Societies for Experimental Biology.

[11] Vila M (2001). Bax ablation prevents dopaminergic neurodegeneration in the 1-methyl- 4-phenyl-1,2,3,6-tetrahydropyridine mouse model of Parkinson’s disease. Proc Natl Acad Sci USA.

[12] Wang DB (2014). Bax interacting factor-1 promotes survival and mitochondrial elongation in neurons. J Neurosci.

[13] Jantas D, Lason W (2009). Protective effect of memantine against Doxorubicin toxicity in primary neuronal cell cultures: influence a development stage. Neurotoxicity research.

[14] Wetzel M, Tibbitts J, Rosenberg GA, Cunningham LA (2004). Vulnerability of mouse cortical neurons to doxorubicin-induced apoptosis is strain-dependent and is correlated with mRNAs encoding Fas, Fas-Ligand, and metalloproteinases. Apoptosis: an international journal on programmed cell death.

[15] Nagai M (2007). Astrocytes expressing ALS-linked mutated SOD1 release factors selectively toxic to motor neurons. Nature neuroscience.

[16] Becattini, B. *et al*. Targeting apoptosis via chemical design: inhibition of bid-induced cell death by small organic molecules. *Chem Biol* **11**, 1107–1117, 10.1016/j.chembiol.2004.05.022 S1074552104001917 [pii] (2004). 10.1016/j.chembiol.2004.05.022 15324812

[17] Civiero L (2012). Biochemical characterization of highly purified leucine-rich repeat kinases 1 and 2 demonstrates formation of homodimers. PLoS One.

[18] Greggio E (2008). The Parkinson disease-associated leucine-rich repeat kinase 2 (LRRK2) is a dimer that undergoes intramolecular autophosphorylation. J Biol Chem.

[19] Miyatake H (2015). Crystal structure of human importin-alpha1 (Rch1), revealing a potential autoinhibition mode involving homodimerization. PLoS One.

[20] Cardona F, Tormos-Perez M, Perez-Tur J (2014). Structural and functional in silico analysis of LRRK2 missense substitutions. Molecular biology reports.

[21] Guaitoli G (2016). Structural model of the dimeric Parkinson’s protein LRRK2 reveals a compact architecture involving distant interdomain contacts. Proc Natl Acad Sci USA.

[22] Lu B (2010). Expression, purification and preliminary biochemical studies of the N-terminal domain of leucine-rich repeat kinase 2. Biochim Biophys Acta.

[23] Sandu C (2006). FADD self-association is required for stable interaction with an activated death receptor. Cell Death Differ.

[24] Xiromerisiou G (2007). Screening for SNCA and LRRK2 mutations in Greek sporadic and autosomal dominant Parkinson’s disease: identification of two novel LRRK2 variants. European journal of neurology: the official journal of the European Federation of Neurological Societies.

[25] Riley JS, Malik A, Holohan C, Longley DB (2015). DED or alive: assembly and regulation of the death effector domain complexes. Cell death & disease.

[26] Carrington PE (2006). The structure of FADD and its mode of interaction with procaspase-8. Molecular cell.

[27] Eberstadt M (1998). NMR structure and mutagenesis of the FADD (Mort1) death-effector domain. Nature.

[28] Kaufmann M (2002). Identification of a basic surface area of the FADD death effector domain critical for apoptotic signaling. FEBS letters.

[29] Kodiha M, Tran D, Morogan A, Qian C, Stochaj U (2009). Dissecting the signaling events that impact classical nuclear import and target nuclear transport factors. PLoS One.

[30] Liu Z (2011). The kinase LRRK2 is a regulator of the transcription factor NFAT that modulates the severity of inflammatory bowel disease. Nature immunology.

[31] Park S (2016). Interplay between Leucine-Rich Repeat Kinase 2 (LRRK2) and p62/SQSTM-1 in Selective Autophagy. PLoS One.

[32] Gomez-Angelats M, Cidlowski JA (2003). Molecular evidence for the nuclear localization of FADD. Cell Death Differ.

[33] Smith WW (2006). Kinase activity of mutant LRRK2 mediates neuronal toxicity. Nature neuroscience.

[34] Herzig MC (2011). LRRK2 protein levels are determined by kinase function and are crucial for kidney and lung homeostasis in mice. Hum Mol Genet.

[35] Steger, M. *et al*. Phosphoproteomics reveals that Parkinson’s disease kinase LRRK2 regulates a subset of Rab GTPases. *eLife* **5**, 10.7554/eLife.12813 (2016). 10.7554/eLife.12813 PMC4769169 26824392

[36] Kantari C, Walczak H (2011). Caspase-8 and bid: caught in the act between death receptors and mitochondria. Biochim Biophys Acta.

[37] Raemy E, Martinou JC (2014). Involvement of cardiolipin in tBID-induced activation of BAX during apoptosis. Chemistry and physics of lipids.

[38] Ho DH (2015). Leucine-Rich Repeat Kinase 2 (LRRK2) phosphorylates p53 and induces p21(WAF1/CIP1) expression. Molecular brain.

[39] Cui J, Yu M, Niu J, Yue Z, Xu Z (2011). Expression of leucine-rich repeat kinase 2 (LRRK2) inhibits the processing of uMtCK to induce cell death in a cell culture model system. Bioscience reports.

[40] Greggio E (2007). Mutations in LRRK2/dardarin associated with Parkinson disease are more toxic than equivalent mutations in the homologous kinase LRRK1. Journal of neurochemistry.

[41] Gahl RF, Dwivedi P, Tjandra N (2016). Bcl-2 proteins bid and bax form a network to permeabilize the mitochondria at the onset of apoptosis. Cell death & disease.

[42] Rideout HJ, Stefanis L (2002). Proteasomal inhibition-induced inclusion formation and death in cortical neurons require transcription and ubiquitination. Molecular and cellular neurosciences.

[43] Perez D, White E (1998). E1B 19K inhibits Fas-mediated apoptosis through FADD-dependent sequestration of FLICE. The Journal of cell biology.

[44] Siegel RM (1998). Death-effector filaments: novel cytoplasmic structures that recruit caspases and trigger apoptosis. The Journal of cell biology.

[45] Scott FL (2009). The Fas-FADD death domain complex structure unravels signalling by receptor clustering. Nature.

