# Supplementary material for: A motif within the armadillo repeat of Parkinson’s-linked LRRK2 interacts with FADD to hijack the extrinsic death pathway
Source: Sci Rep. 2018 Feb 22;8:3455. doi: 10.1038/s41598-018-21931-8 (PMC5823876; doi:10.1038/s41598-018-21931-8)
Supplement: Supplementary file 1 — Supplementary Data [file 41598_2018_21931_MOESM1_ESM.pdf]

A motif within the armadillo repeat of Parkinson's-linked LRRK2 interacts with FADD to hijack the extrinsic death pathway

Nasia Antoniou[a]; Dimitrios Vlachakis[b]; Anna Memou[a]; Emmanouela Leandrou[a]; Polytimi-Eleni Valkimadi[a]; Katerina Melachroinou[a]; Diane B. Re[c]; Serge Przedborski[d]; William T. Dauer[e]; Leonidas Stefanis[a,f]; *Hardy J. Rideout[a]

Supplementary Information.

Supplementary Figure 1. Representative confocal images of primary embryonic cortical neurons transiently expressing mutant R1441C or I2020T-LRRK2 and treated with vehicle control, or inhibitors of translocation of Bax to mitochondria (V5), or translocation of truncated Bid (BI-6C9) to mitochondria. Neurons were fixed and processed for double immunofluorescence labeling with anti-EGFP and anti-LRRK2 (clone c41-2; red channel), with DAPI as a nuclear counter stain. Images were acquired using a Leica TSP5 multi-photon confocal microscope. Asterisks indicate LRRK2 expressing neurons.

Supplementary Figure 2. Ramachandran plots and Procheck evaluation of the LRRK2 model in comparison to its template structure (human Importin-alpha1/rch1, PDB id: 3WPT). The Procheck summary (lower Table) refers to the final model of the LRRK2 ARM repeat region upon energy minimization and molecular dynamics optimizations.

Supplementary Figure 3. The FADD DD/Fas crystal structure (PDB id: 3EZQ). The α-helix of Fas (blue ribbon) is displayed with its corresponding α-helix interacting segment of FADD DD (magenta ribbon). The Fas fragment was aligned against the LRRK2 (orange ribbon) interacting region (residues 532-547) of LRRK2.

Supplementary Figure 4. Electro static map for the FADD-bound LRRK2 model. The LRRK2 ARM model has an overall neutral charge, with no heavily charged regions. The colour conventions for the displayed model are identical to those of Figures 4-6 of the main text.

Supplementary Figure 5. Optimization of the docked LRRK2 / FADD DD complex. The docked complexes were solvated in a water filled periodic cell for all subsequent energy minimization and molecular dynamics simulations.

Supplementary Figure 6. The molecular dynamics trajectory of the full LRRK2 dimer molecular system, in the presence of the four docked FADD molecules.

Supplementary Figure 7. The *in silico* modeled interaction site between LRRK2 and FADD DD. (a) The interaction part of the model between LRRK2 and FADD. LRRK2 is shown in orange ribbon and FADD in purple. (b) The molecular interactions in the LRRK2 / FADD complex. (c) Electrostatic surface analysis of the interaction (d) Pocket complementarity analysis of the interaction.

Supplementary Figure 8. Molecular dynamics of the K544E mutant LRRK2 and FADD-DD. (a) The original model of monomeric LRRK2 was used to estimate the molecular interactions of the 544 Lysine residue. (b) The K544E mutation was induced and the molecular interactions of the introduced Glutamic acid were calculated after energy minimization and molecular dynamics. (c) The molecular dynamics trajectory charts of the WT and the K544E molecular systems.

Supplementary Figure 9. LRRK2 fails to interact with monomeric FADD. HEK293T cells were co-transfected with either Flag-tagged WT or G2019S-LRRK2 and V5-tagged WT or F25R mutant FADD. Parallel cells were co-transfected with G2019S-LRRK2 and full-length FADD plus HA-tagged dimeric FADD-DD. While WT FADD binds WT-LRRK2 and more strongly to G2019S-LRRK2; F25R-FADD, which cannot self-associate, is unable to bind either WT or G2019S mutant LRRK2. Similarly, the dominant negative dimeric FADD-DD (lzDD), blocks the interaction between G2019S mutant LRRK2 and FADD.

Supplementary Figure 10. Deletion of predicted FADD-binding domain in LRRK2 N-terminal blocks co-localization with FADD. HEK293T cells were transiently co-transfected with V5-FADD and Flag-LRRK2 (G2019S, a; or I2020T, b). Cells were fixed and processed

for anti-V5 (red) and anti-Flag (green). Note the lack of co-localization with FADD-positive DEFs in cells expressing LRRK2 lacking the FADD binding domain.

Supplementary Figure 11. N-terminal fragments of LRRK2 containing the FADD binding domain are neuroprotective against mutant LRRK2 induced neuronal death. Primary rat embryonic cortical neurons were transiently transfected with full-length mutant (R1441C, G2019S, or I2020T) LRRK2 together with fragments of the LRRK2 N-terminal that fail to bind FADD (NT500) or bind strongly to FADD (NT575). The neurons were fixed 72h later and processed for double immunofluorescence for anti-LRRK2 (clone c41-2; red) and EGFP, and the nuclear stain DAPI. Representative images were acquired using a Leica TSP5 multi-photon confocal microscope. Note the preservation of nuclear morphology indicating surviving neurons in cultures co-transfected with NT575. Asterisks indicate LRRK2 expressing neurons.

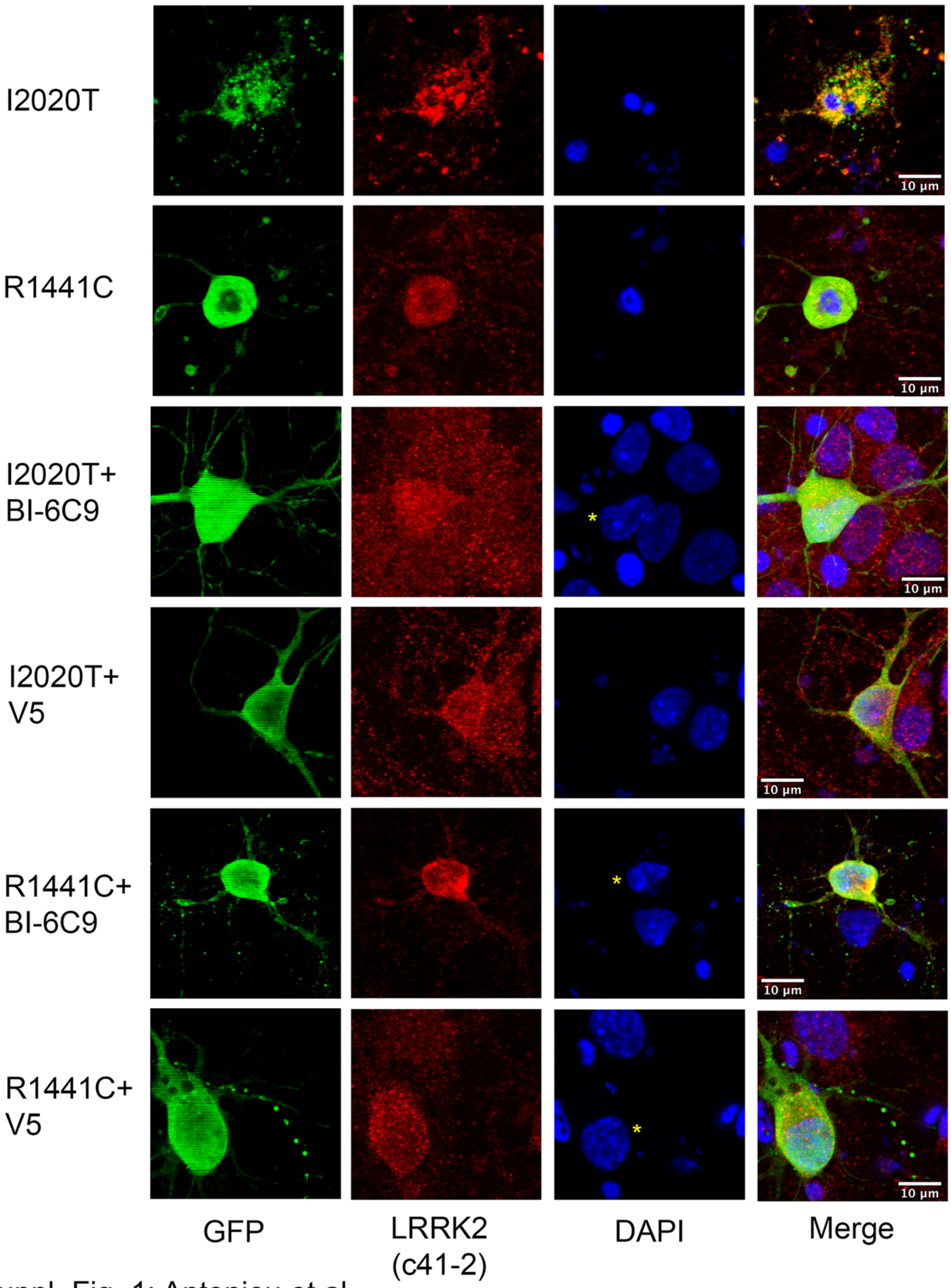

Suppl. Fig. 1; Antoniou et al.

TEMPLATE MODEL

Ramachandran Plot

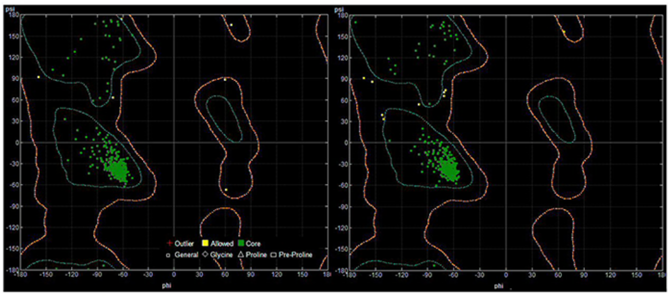

PROCHECK statistics

1. Ramachandran Plot statistics

		%-tage of residues
Most favoured regions	[A,B,L]	97.5%
Additional allowed regions	[a,b,l,p]	2.5%
Generously allowed regions	[~a,~b,~l,~p]	0.0%
Disallowed regions	[XX]	0.0%
Non-glycine and non-proline residues		100.0%

Suppl. Fig.2; Antoniou et al.

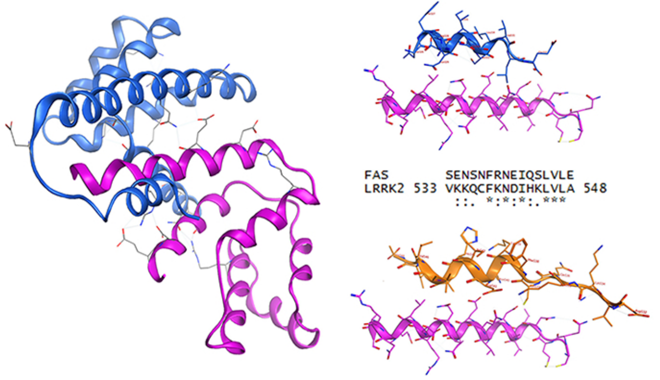

Suppl. Fig. 3; Antoniou et al.

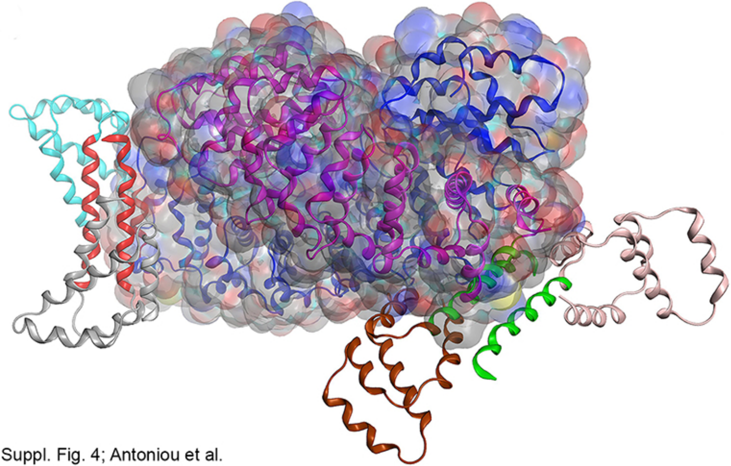

Suppl. Fig. 4; Antoniou et al.

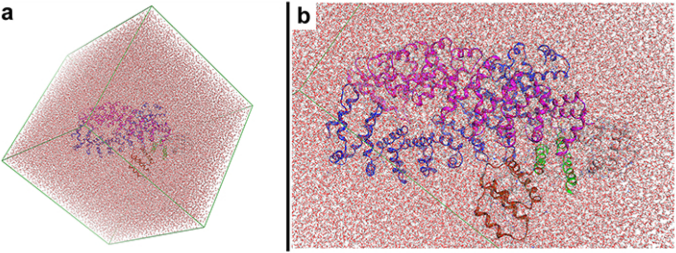

Suppl. Fig. 5; Antoniou et al.

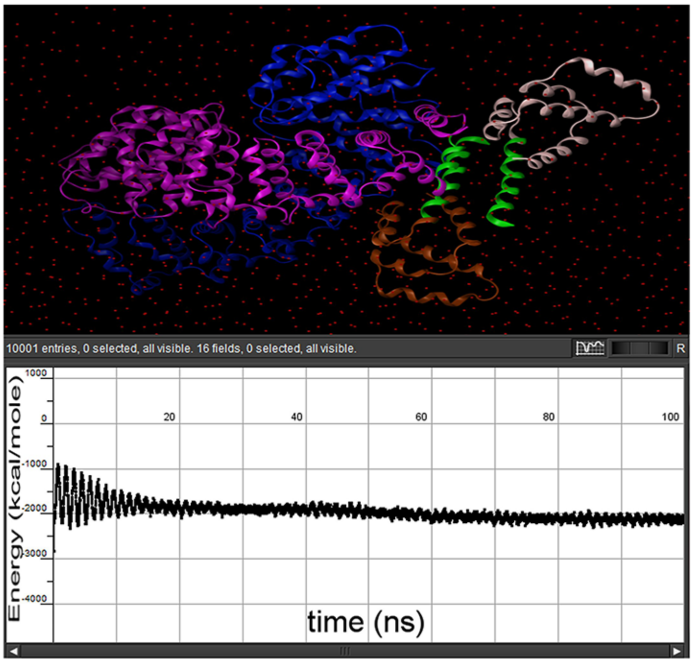

Suppl. Fig. 6; Antoniou et al.

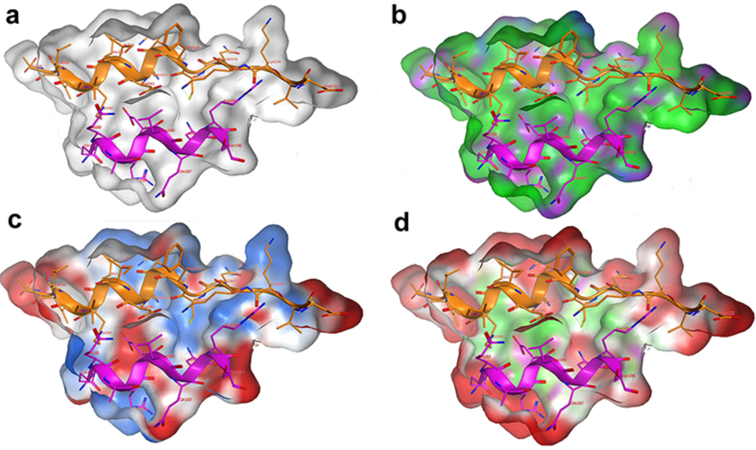

Suppl. Fig. 7; Antoniou et al.

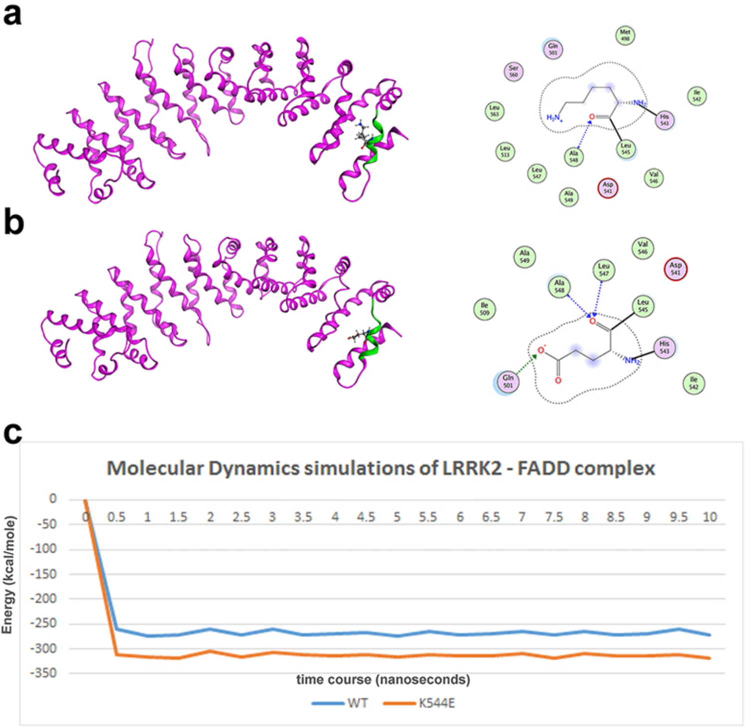

Suppl. Fig. 8; Antoniou et al.

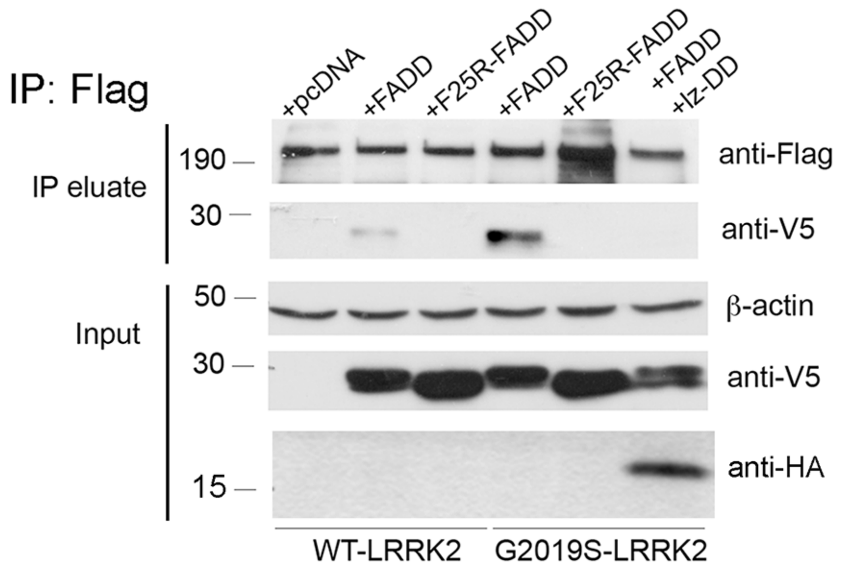

Suppl. Fig 9; Antoniou et al.

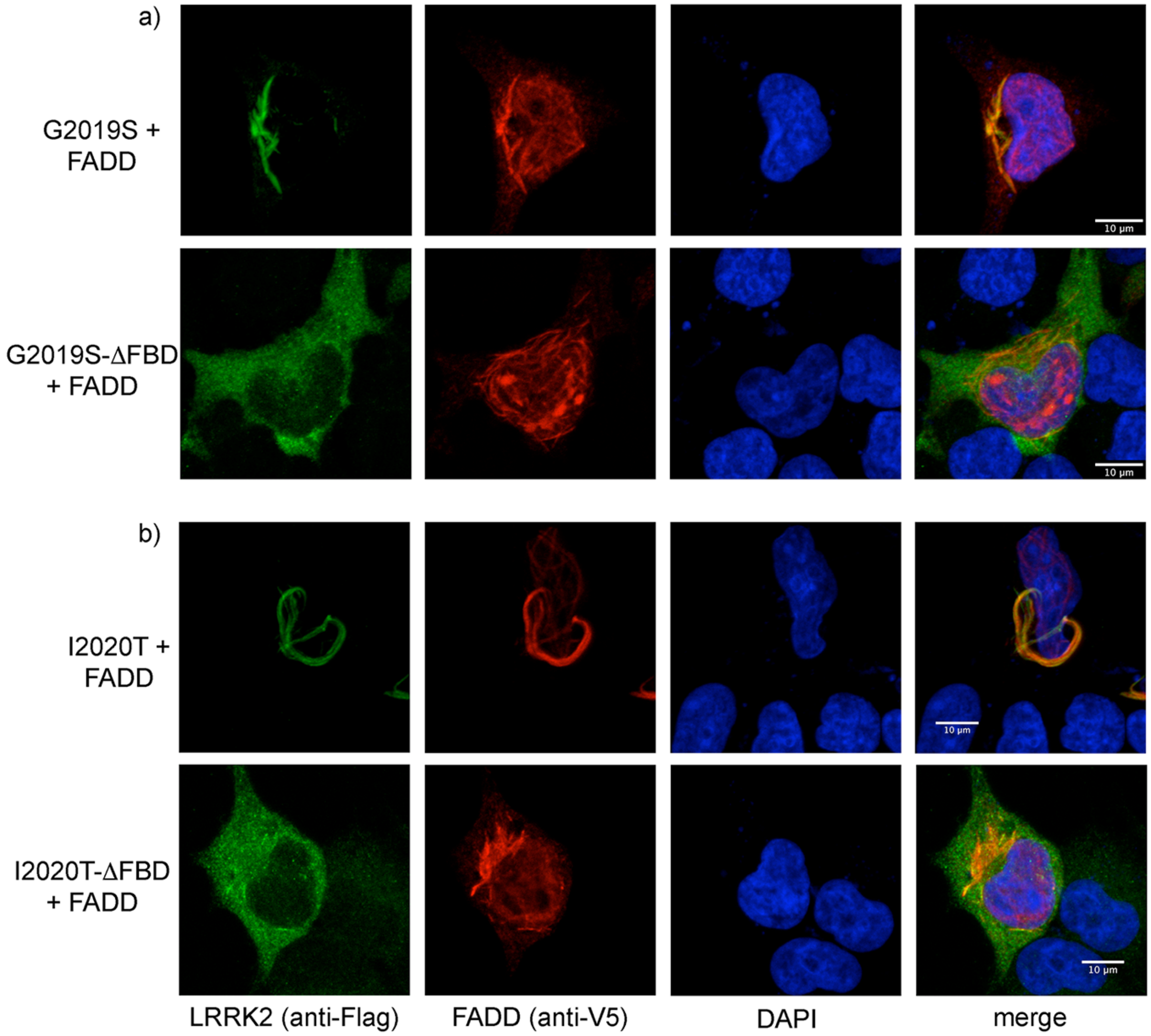

Suppl. Fig. 10; Antoniou et al.

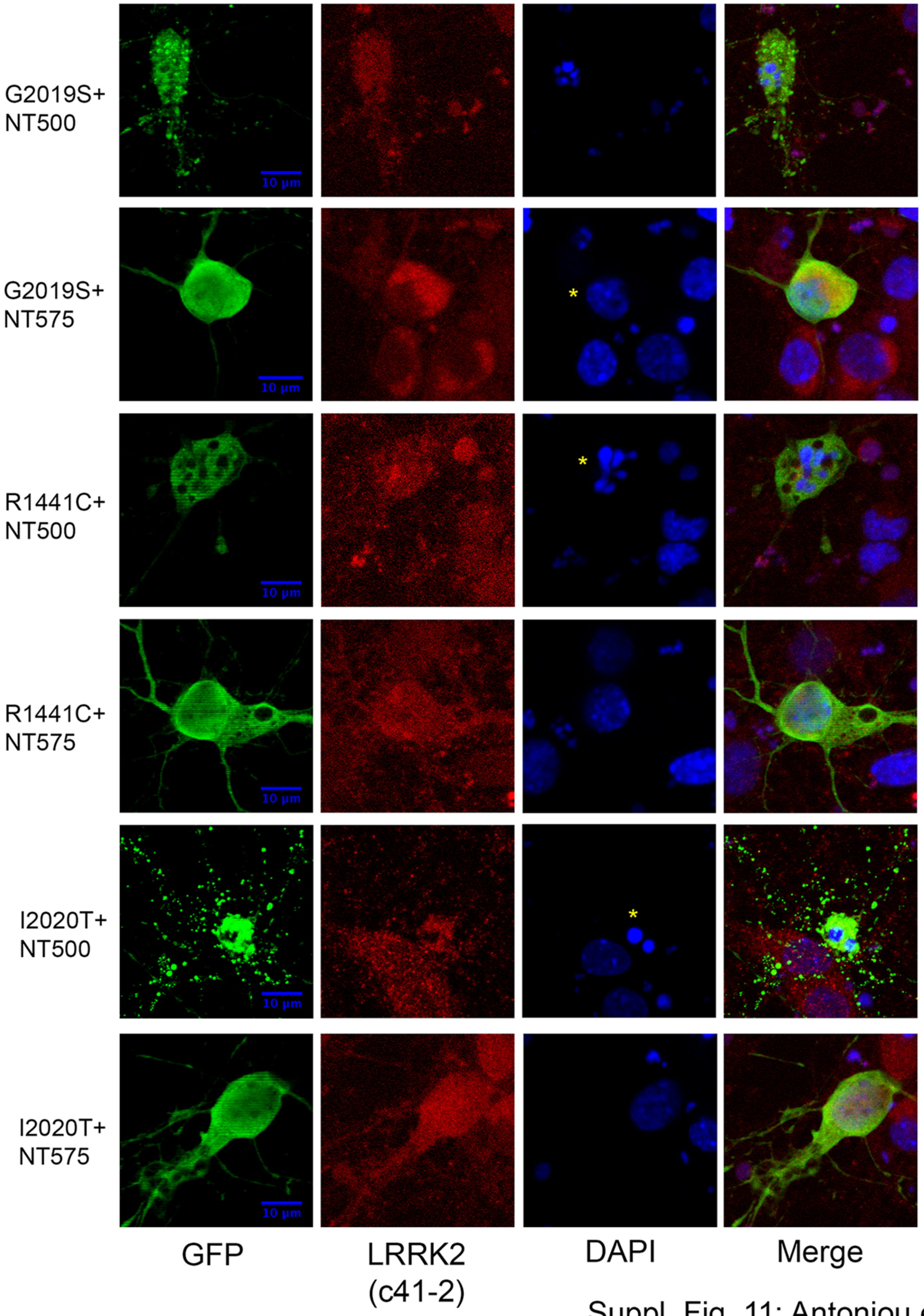

Suppl. Fig. 11; Antoniou et al.
